# Ten strategies for a successful transition to remote learning: Lessons learned with a flipped course

**DOI:** 10.1002/ece3.6760

**Published:** 2020-10-16

**Authors:** Ana E. Garcia‐Vedrenne, Chloé Orland, Kimberly M. Ballare, Beth Shapiro, Robert K. Wayne

**Affiliations:** ^1^ Department of Ecology and Evolutionary Biology University of California Los Angeles Los Angeles CA USA; ^2^ Department of Ecology and Evolutionary Biology University of California Santa Cruz Santa Cruz CA USA; ^3^ Howard Hughes Medical Institute University of California Santa Cruz Santa Cruz CA USA

**Keywords:** active learning, flipped classroom, inclusivity, remote instruction

## Abstract

Transitioning from in‐person to remote learning can present challenges for both the instructional team and the students. Here, we use our course “Biodiversity in the Age of Humans” to describe how we adapted tools and strategies designed for a flipped classroom to a remote learning format. Using anonymous survey data collected from students who attended the course either in‐person (2019) or remotely (2020), we quantify student expectations and experiences and compare these between years. We summarize our experience and provide ten “tips” or recommendations for a transition to remote learning, which we divide into three categories: (a) precourse instructor preparation; (b) outside of class use of online materials; and (c) during class student engagement. The survey results indicated no negative impact on student learning during the remote course compared to in‐person instruction. We found that communicating with students and assessing specific needs, such as access to technology, and being flexible with the structure of the course, simplified the transition to remote instruction. We also found that short, pre‐recorded videos that introduce subject materials were among the most valuable elements for student learning. We hope that instructors of undergraduate ecology and evolution courses can use these recommendations to help establish inclusive online learning communities that empower students to acquire conceptual knowledge and develop scientific inquiry and literacy skills.

## Graphical abstract

Transitioning from in‐person to remote learning can present challenges for both the instructional team and the students. Here, we use our course “Biodiversity in the Age of Humans” to describe how we adapted tools and strategies designed for a flipped classroom to remote learning. We provide ten “tips,” which we divide into three categories: (a) precourse instructor preparation; (b) outside of class use of online materials; and (c) during class student engagement. We hope that instructors of undergraduate ecology and evolution courses can use these recommendations to help establish inclusive online learning communities that empower students.

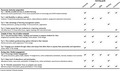

## 1 INTRODUCTION

A key step in supporting our students and addressing problems of student persistence and retention in STEM fields lies in shifting the focus from “what” we teach to “how” we teach it. Student success and persistence in STEM are known to increase when (a) active‐learning strategies are used effectively in the classroom (Freeman et al., 2014; Haak, HilleRisLambers, Pitre, & Freeman, 2011), and (b) students are exposed to authentic research experiences during the first few years of undergraduate education (Eagan et al., 2013; Laursen, Hunter, Seymour, Thiry, & Melton, 2010; Olson & Riordan, 2012; Russell, Hancock, & McCullough, 2007).

The logistics associated with designing such learning environments may seem complicated given limited availability of funds, resources, space, and mentors, even under ‘normal’ circumstances. Recent events related to the COVID‐19 pandemic have amplified these problems as schools and universities transition to remote instruction, for example by amplifying existing structural disparities and creating more obstacles to offering authentic research experiences to undergraduate students. Despite well‐intended efforts to provide quality instruction, many professors and students have struggled with the transition to giving and receiving instruction outside of the classroom and online, encountering hardships including limited access to reliable Internet and technology, lack of familial support, lack of private or designated space for learning free from distractions, and a lack of professional training in how to provide or participate in remote learning.

Despite these challenges, public health concerns require students and instructors to remain off‐campus at most colleges and universities, necessitating online learning alternatives. As virtual learning becomes more common, it is important to adapt our teaching strategies to acknowledge this new reality and to identify ways to best serve our students with this form of teaching engagement.

**FIGURE 1:**
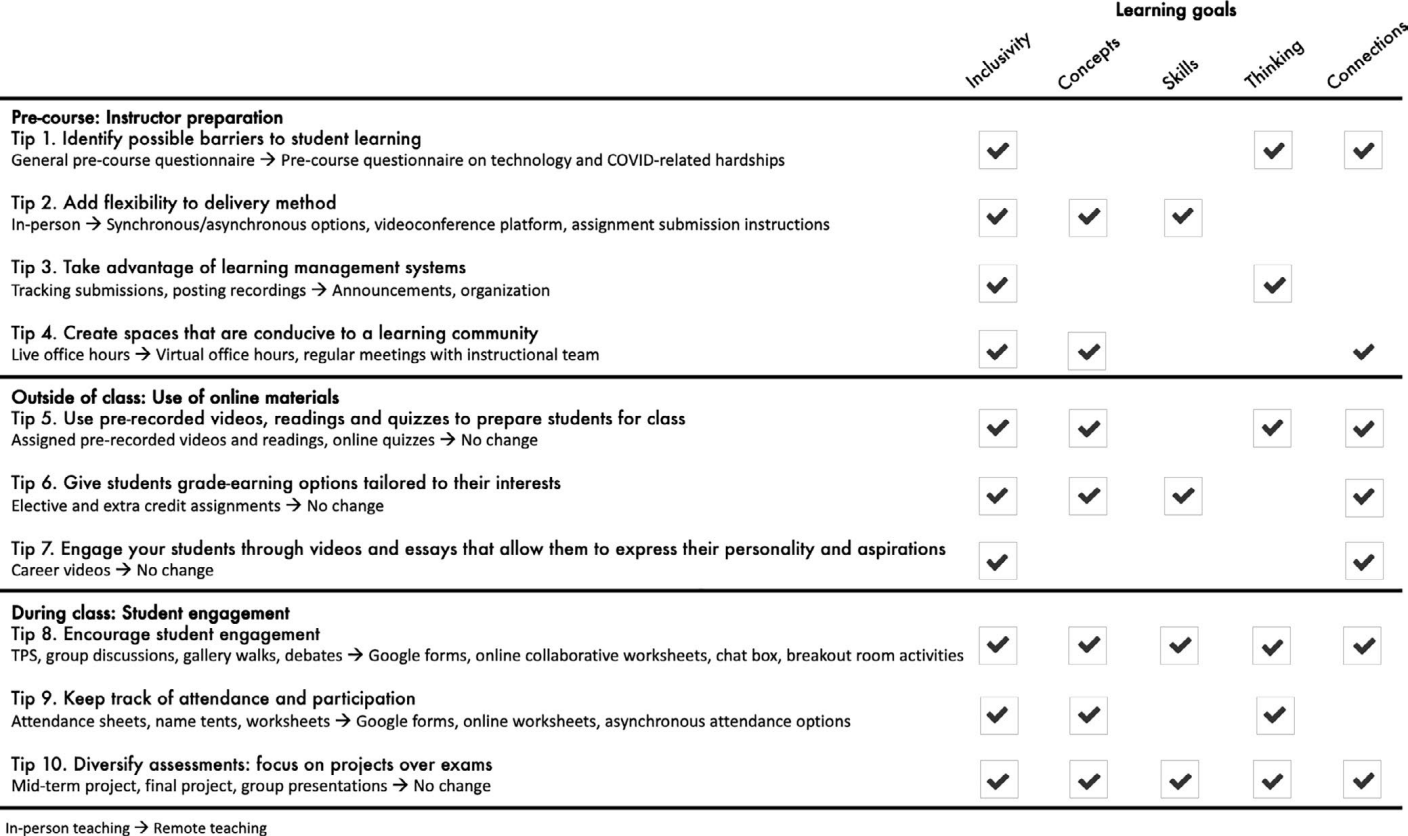
Ten tips on how to transition from an in‐person to a remote classroom, with specific examples of how we implemented these changes in our course (listed below each tip). Arrows indicate how the tools and activities were adapted from in‐person instruction to remote format. “No change” indicates that the in‐person activities created for the in‐person flipped classroom transitioned easily between in‐person and remote formats. A summary of the learning goals achieved by each tip is indicated by checkmarks in adjacent columns. Inclusivity: create a course that is inclusive and fair for all students; concepts: foster an environment that empowers students to acquire conceptual knowledge; skills: develop scientific inquiry and literacy skills; thinking: provide students time to think about class materials and be primed to participate; and connections: encourage students to reflect on personal connections with biology and their desired career path. TPS, think‐pair‐share

Although adapting in‐person undergraduate courses to remote learning in a short period of time may seem intimidating and overwhelming, many teaching strategies, tools, and technologies are already available, some of which can be adopted quickly and with minimal assistance from support staff. Here, we describe how we adapted tools and strategies designed for an active‐learning classroom to a remote learning experience, and provide recommendations for their use in Ecology and Evolution undergraduate courses. Specifically, we present our introductory biology course “Biodiversity in the Age of Humans” as a case study and describe the tools that we found to be effective to preserve the learning goals originally designed for this course as an in‐person, flipped classroom. Our objectives were to (a) create a course that is inclusive and fair for all students; (b) foster an environment that empowers students to acquire conceptual knowledge; (c) develop scientific inquiry and literacy skills; (d) provide students time to think about class materials and be primed to participate; and (e) encourage students to reflect on personal connections with biology and their desired career path. Here, we quantify student expectations and experiences from anonymous survey data collected during both in‐person (2019) and remote teaching (2020) and compare these results within and between years. We then provide 10 recommendations for a transition to remote learning (“Tips,” Figure 1) and divide these tips into three categories: (1) precourse instructor preparation; (2) outside of class use of online materials; and (3) during class student engagement.

### 1.1 “Biodiversity in the Age of Humans”—A case study

The “Biodiversity in the Age of Humans” course is an undergraduate introductory biology class taught concurrently at University of California Los Angeles (UCLA) and UC Santa Cruz (UCSC). The course explores environmental DNA (eDNA; DNA from the environment that can provide information on the species present at a locality) as a tool to explore scientific questions about biodiversity worldwide. The curriculum includes measuring past and present biodiversity using eDNA, understanding ethics in research, learning approaches to DNA sequencing and analysis, and developing connections between biodiversity, human health, and ecosystem health. Within these topics, the course material emphasizes professional development skills as students learn to use the scientific method, ask and answer questions about eDNA, search and use the scientific literature, write scientific content, and develop science communication skills (Figures S1 and S2).

“Biodiversity in the Age of Humans” is a class in the Ecology and Evolutionary Biology department that enrolls up to 70 students at each UC campus. There are no prerequisites to taking this course and it is open to students from all majors and disciplines. The course was originally designed to target new students (first year, second year, and transfer students), but was open to undergraduates of all standings in both 2019 and 2020. In 2019, 65 students enrolled in the course (35 from UCLA and 30 from UCSC); in 2020, 104 students were enrolled (65 from UCLA and 39 from UCSC; see Table 1 for enrollment data).

**FIGURE 2:**
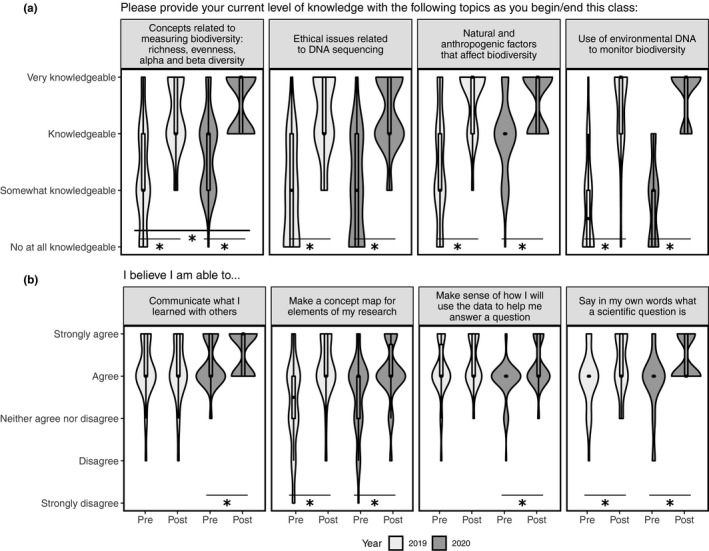
(a) Student self‐assessed level of knowledge about select course topics before the course started (pre) and at the end of the course (post) for the in‐person 2019 course (*n* = 30) and the remote 2020 course (*n* = 50). The survey provided further clarification regarding the knowledge categories as follows: (1) Not at all knowledgeable (i.e., I am unfamiliar with the topic); (2) somewhat knowledgeable (i.e., I have heard of the topic but could not readily explain it to someone); (3) knowledgeable (i.e., I have heard of the topic and could readily explain what it means to someone); and (4) very knowledgeable (i.e., I understand current research on the topic and could teach it to a peer). Only select topics are shown (see Figure S1 for full dataset). (b) Student self‐assessed ability to perform inquiry skills at the beginning of the course (pre) and at the end of the course (post) for the in‐person 2019 course (*n* = 30) and the remote 2020 course (*n* = 50). Only select skills are shown (see Figure S2 for full dataset). *Topics and skills where the change in perceived knowledge or ability is significant (*p* < .05) before and after the course as indicated by paired *t* tests

**FIGURE 3:**
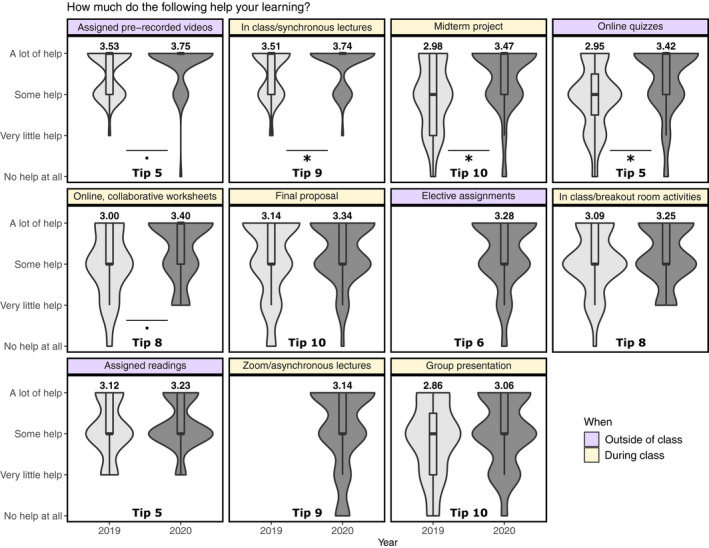
Student‐perceived usefulness of various activities and strategies implemented throughout the in‐person 2019 course (n = 43) and adapted for the remote 2020 course (n = 53). Responses have been organized from most helpful to least helpful as indicated by student responses in the postcourse survey following the 2020 remote course. Means are derived from Likert scale values: (1) no help at all; (2) very little help; (3) some help; and (4) a lot of help. Although elective assignments were a part of the 2019 course, these were not entries in our postcourse survey. Asynchronous recording of lectures was only offered in 2020 for students unable to join synchronously. The number of the tip that the activity relates to is indicated at the bottom center of each box. Outside of class tips (Tips 5–7) are highlighted in purple; during class tips (Tips 8–10) are highlighted in yellow. Asterisks (*) and dots (.) indicate activities where the perceived change in usefulness is significant (p < .05) or close to significant (p < .07) between 2019 and 2020

**FIGURE 4:**
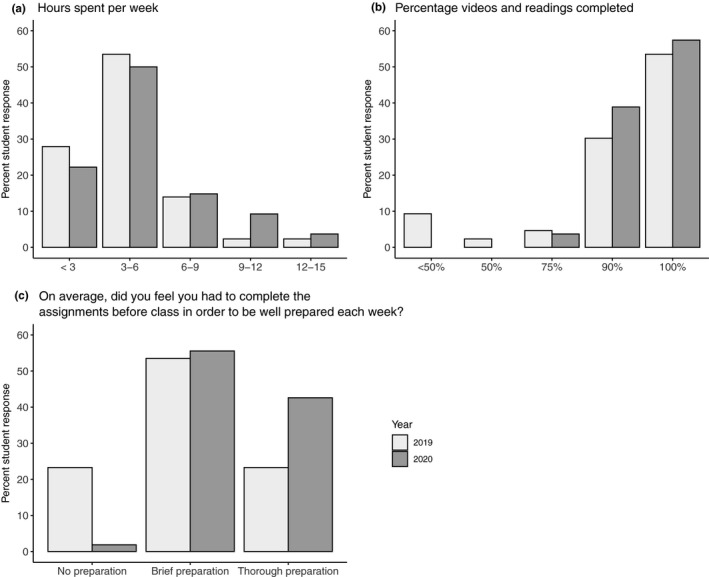
Bar plots show student responses to the following prompts: (a) Approximately how many hours per week, outside of regular class meetings, did you spend on this class? (b) About what percentage of the assigned readings and videos did you complete? (c) On average, did you feel you had to complete the assignments before class in order to be well prepared each week? Only (c) significantly differed between years as determined by *χ* ^2^(2) = 12.61; *p* = .0018. For (a, b, and c), 2019: *n* = 43; 2020: *n* = 54

We conducted anonymous student surveys to assess student learning expectations and outcomes in both 2019 and 2020. Survey data were collected at two time points, at the beginning (“pre‐course”) and end of the quarter (“post‐course”). Response rates were 71% for the precourse surveys and 62% for postcourse surveys. In 2019, 33 students answered both pre‐ and postsurveys (51%) and 51 students answered both surveys in 2020 (49%). Although the survey instruments were not identical between years, most questions did overlap or have an equivalent question across years. Our surveys focused on (1) student self‐assessed level of knowledge about course topics before the course started (pre) and at the end of the course (post) (Figure 2a); (2) student self‐assessed ability to perform inquiry skills at the beginning of the course (pre) and at the end of the course (post) (Figure 2b); (3) student‐perceived usefulness of various activities and strategies implemented throughout the in‐person 2019 course and adapted for the remote 2020 course (Figure 3); and (4) information on time spent working on class activities, degree of completion and perceived need to complete assignments (Figure 4). In addition, our postcourse survey included open‐ended questions about student opinions on course format and activities.

**TABLE 1:** Demographic information about our courses in 2019 (*n* = 65) and 2020 (*n* = 104)

	UCSC			UCLA		
	2019	2020	Campus‐wide	2019	2020	Campus‐wide
	(*n* = 30)	(*n* = 39)	(*n* = 17,517)	(*n* = 35)	(*n* = 65)	(*n* = 31,577)
Gender (%)						
F	46.7	51.3	47.5	57.1	60.9	42
M	43.3	35.9	51.6	42.9	39.1	58
U/X	1.0	12.8	0.9	NA	NA	NA
Pell grant recipient (%)	38	29	36	40	35.9	35
First generation (%)	36.7	23.1	37	31.4	18.8	32
URM (%)	33.3	25.6	30.4	45.7	29.7	26
Transfer (%)	13.3	2.6	20.2	34.3	7.8	25.9

Note

Campus‐wide undergraduate student enrollment data were collected in Fall 2018 at UCLA and in Fall 2019 at UCSC. Pell Grants are awarded to students who display exceptional financial need. Students are considered to be first generation when their parents have not graduated from a four‐year college or university. Underrepresented minorities (URM) include the following race/ethnicities: Black/African American, Hispanic/Latino/a and Native American. F, female, M, male, U, undisclosed, X, unspecified.

We performed statistical tests in the JMP software package (JMP^®^, version 14; SAS Institute Inc., 1989–2019). We used paired *t* tests to evaluate whether students' self‐assessed level of knowledge about course topics and ability to perform inquiry skills had changed as a result of the course. We then performed a Welch *t* test on the differences between paired pre‐ and postsurvey results to see whether this perceived gain varied between the 2019 in‐person format and the 2020 remote format. A Welch *t* test was also conducted to determine whether student‐perceived usefulness of activities changed between 2019 and 2020. Finally, we performed chi‐square tests on responses related to (a) time spent working on class activities; (b) degree of completion; and (c) perceived need to complete activities to evaluate whether these had been different in 2019 and 2020.

#### 1.1.1 Survey findings

First, we analyzed students' self‐assessed level of knowledge about course topics and their ability to perform scientific inquiry and literacy skills. Specifically, we tested whether there had been any change between the knowledge and skill level they reported at the beginning of the course and the level they reported achieving as a consequence of completing the course (Figure 2; Figures S1 and S2). The change in perceived knowledge of the topics was significant as indicated by paired *t* tests (*p* < .05) for all topics in both 2019 and 2020 (Figure 2a; Figure S1). The change in perceived ability to perform scientific skills was significant for some skills, including skills related to research ability and scientific inquiry (Figure 2b; Figure S2). Students reported perceived increases in ability across more skills in 2020 (18 out of 20) than in 2019 (4 out of 20; Figure S2). We then tested whether this perceived change in knowledge and abilities was different between 2019 and 2020. Perceived change in knowledge did not significantly vary between 2019 and 2020, except for “Concepts related to measuring biodiversity such as richness, evenness, alpha and beta diversity” which was greater in 2020 (*t*(48.2) = −2.57; *p* = .0133; Figure 2a; Figure S1). Perceived change in skills did not vary significantly between 2019 and 2020, except for the skill “Look for information beyond textbooks,” which was also higher in 2020 (*t*(56.3) 2.44; *p* = .0176; Figure S2).

Second, we analyzed students' ratings of how activities and strategies implemented throughout the in‐person 2019 course and the remote 2020 course impacted their learning (Figure 3). In both 2019 and 2020, students reported that pre‐recorded videos and in‐class or synchronous lectures were the elements that contributed most to their learning. Students in 2020 indicated that activities such as the midterm project, online quizzes, and collaborative worksheets were particularly helpful compared to 2019 (*p* < .07; Figure 3). We found no change between years in perceived usefulness of the other activities we implemented (Figure 3).

Most students participated synchronously, with attendance averaging 80% (range 77%–84%). 35/104 students attended at least one asynchronous lecture. Of those, 22 used the asynchronous option only once or twice in the quarter. Overall, students felt that real‐time interaction with instructors was important to their learning (Figure 3), but appreciated the flexibility of having both synchronous and asynchronous options for attending lecture. The “No help at all” and “Very little help” ratings of asynchronous lectures (Figure 3) may reflect that some students never attended asynchronously, as our survey did not provide an N/A option.

Finally, we compared the self‐reported level of effort invested by our students between the two offerings. Most students reported spending between three and six hours each week on course‐related activities, and the time spent on course‐related activities outside of class time did not change between years (Figure 4a). The majority of students reported completing between 90%–100% of preclass assignments, with no change between years (Figure 4b). Although the time that students reported spending on course‐related activities outside of class time was similar across years, students reported a perceived need to be better prepared for the remote class than for the in‐person course (*χ* ^2^(2) = 12.61, *p* = .002; Figure 4c).

Overall, these survey results indicate little difference between the 2019 in‐person and 2020 remote offering of our course in the extent to which our desired learning outcomes were achieved. We acknowledge that our sample size is small and we are only able to compare data from single years. However, we believe these trends nonetheless provide some insight into successful strategies to create valuable remote learning experiences.

Below, we summarize our experience and present 10 tips or recommendations for a transition to remote learning that worked well in our experience (Figure 1). We hope that instructors of undergraduate ecology and evolution courses can implement these recommendations to help establish inclusive online learning communities, empowering students to acquire conceptual knowledge and develop scientific inquiry and literacy skills.

## 2 ADAPTING AN ACTIVE‐LEARNING FLIPPED CLASSROOM TO REMOTE LEARNING: TIPS FOR A SMOOTH TRANSITION

### 2.1 Precourse: Plan for both structure and flexibility

Transferring from an in‐person to a remote class brings an entire new set of challenges for both the instructional team and the students. We found that communicating with students and assessing their needs were essential factors to ease this transition. Creating a syllabus that incorporated flexibility was also important for us to meet the needs of the majority of students as the pandemic situation continued to shift day‐to‐day operations and priorities. Of all the tips we provide in this paper, we believe that those given in this first section on precourse adjustments are particularly important to implement and would be effective to retain if transitioning back to in‐person teaching.

#### 2.1.1 Tip 1. Identify possible barriers to student learning

In both 2019 and 2020, we contacted students prior to the start of the course via an introductory email and precourse questionnaire. Questionnaire responses may provide important information regarding course demographics, student prior knowledge, interests, and/or needs. This information assists in designing the format of the course and tailoring to student background, interests, and expectations. Emailing students with a request to fill out the questionnaire also provides an opportunity to write a warm, welcoming message and introduction to both course and instructors. Conveying the message that instructors care about student learning and experiences, and opening a line of communication between students and instructors can be critical in increasing student engagement, motivation, and participation (Rodriguez‐Keyes, Schneider, & Keenan, 2013).

Precourse questionnaires are arguably even more important when implementing a course under extraordinary circumstances such as the COVID‐19 pandemic. In 2020, we added an additional questionnaire to specifically assess student concerns, challenges, and potential barriers to remote learning (“Access to Technology Questionnaire,” Appendix S1). For example, we learned that, although the majority of our students would have reliable access to online course materials (reliable high‐speed Internet, computer access) and the ability to attend synchronous lectures, this was not true for everyone. Specifically, several of our students notified us that they would be participating in the course from remote time zones, several did not have reliable Internet, or would be participating in the class with a shared computer or in a nonprivate area of their home.

#### 2.1.2 Tip 2 Add flexibility to delivery method: provide dynamic synchronous and asynchronous options

With the results of the Access to Technology Survey, we were able to alter our syllabus and build flexibility into the remote course prior to the start of the term. Namely, we offered both asynchronous and synchronous options for accessing course content and offered multiple options for completing activities and assignments.

As a flipped classroom, students in our course had flexibility to view preclass video lectures on their own time, but in‐person attendance was required in our 2019 in‐person course. In 2020, we needed to transition to remote learning at short notice, and we brainstormed ways to maintain student engagement while preserving an equitable learning environment. A handful of students were only able to participate asynchronously throughout the course, but we encouraged students to attend synchronously when possible so as to maintain active‐learning goals through group participation in in‐class activities and assignments. Our solution was to provide students with synchronous and asynchronous attendance and participation options (Options “A” and “B,” respectively; see “Syllabus,” Appendix S1; see Tips 2.3.1, 2.3.2). Those students choosing the asynchronous option completed in‐class group assignments individually. Students also had the option to choose Option B either for the entire term or on a class‐to‐class basis. This choice allowed students who wanted to attend synchronously but had occasional barriers to Internet access or other occasional commitments (e.g., conflicting work schedules, caring for family) to do so.

We taught synchronously through video conferencing. Students were required to watch pre‐recorded videos before class (see Tip 5). During class, we typically conducted a short lecture (10–30 min) before beginning any in‐class activities. All synchronous classes were recorded and posted for asynchronous students to view, thus providing opportunities for asynchronous students to engage in active learning throughout the recorded lecture (see Tips 2.3.1, 2.3.2).

The use of video conferencing tools is not new in higher education. Research shows that lectures delivered by videoconference can be as effective as in‐person delivery (Pitcher, Davidson, & Napier, 2000). We decided to use Zoom (https://zoom.us/) given that both UCLA and UCSC provided subscriptions to the “Pro” version, allowing for longer sessions with groups of over 100 participants across multiple campuses, recording of synchronous lectures, and integration within each campus’ learning management system (LMS, see Tip 3). Both pro and free Zoom platforms provide options for mobile and desktop user applications, creation of small groups (“breakout rooms”), and participation tools such as reaction functions (raise hand, clap), classroom polling, real‐time messaging (chat function), attendance tracking, shared whiteboard, and other tools. Although Zoom was likely the most widely used video conferencing system across universities in the United States during remote learning in 2020, some drawbacks include cost of the pro version and security concerns (Marczak & Scott‐Railton, 2020). Other video conferencing platforms include Google Meet, Skype, and Discord, many of which are free to use. Institutions and instructors may wish to explore other technologies to discover what works best for their courses.

#### 2.1.3 Tip 3. Take advantage of learning management systems

Creating synchronous and asynchronous options in 2020 (see Tip 2) was necessary to continue our original course goals of student inclusiveness and active learning (Figure 1). However, this flexibility in delivery inevitably created additional challenges for instructors. For example, instructors had to keep track of attendance and participation of three groups of students (synchronous, full‐time asynchronous, and part‐time asynchronous; see Tip 9). To do so effectively, we created a course website using the university learning management systems (LMS). This provided a centralized location for course materials, communication with students, and records of assessments and grades.

LMS, such as Moodle, Canvas, Blackboard, and Desire2Learn, are increasingly used in higher education, although the ways in which they are used varies by course and institution (Machajewski, Steffen, Fuerte, & Rivera, 2019). As our course was taught at two different institutions that use different integrated platforms, we used two different LMS: Moodle at UCLA and Canvas at UCSC. While we also used these LMS during the in‐person offering in 2019, we found that we relied on LMS even more during the online offering in 2020 to send out announcements and keep track of student assignment submissions, grades, and participation for both synchronous and asynchronous options. We also used the LMS to provide links to activities and assignments that we provided as hard‐copy worksheets during in‐person teaching in 2019.

During the remote offering, we found the LMS useful in particular for those students who were unable to follow the class at the normal pace. Each week was incorporated into the LMS structure as a distinct block or “module,” within which we organized the elements students needed for each day of the class, including assignments, quizzes, and Zoom links. The LMS automatically translated these elements to a task calendar and a day‐to‐day “to‐do list” for students. We also posted recordings of synchronous classes within these modules. In summary, we found that modules were key both to organization, such that students could easily see the assignments and activities that were required, and instructors were able to track student engagement with the course content by visualizing student log‐ins and number of late and on‐time assignment submissions. We note that many LMS are not simple to set up. Therefore, instructors choosing to use a LMS for the first time should liaise with university support staff and/or work through available tutorials to structure course webpages for unique course needs.

A final benefit of LMS that is particularly important during remote learning is the flexible communication options that allow students to communicate with each other and with instructors. For example, although we followed advice to provide a clear, learning‐focused syllabus before the start of the term to guide students as to how to succeed in our course (Palmer, Wheeler, & Aneece, 2016), challenges faced by students and instructors during the spring of 2020 led to a need to change aspects of our grading. We used structures built into the LMS to inform students of any changes to course content and policies, through course‐wide announcements and posted updated syllabi throughout the term. In this way, students were always able to find the most up‐to‐date information about the course by logging into the same location.

#### 2.1.4 Tip 4. Create spaces that are conducive to a learning community

Developing a successful learning community is key to enhancing the learning experience. However, this can be a challenge during remote learning with few, if any, in‐person interactions. Here, we present tools to improve interactions: (a) between instructors; (b) between instructors and students; and (c) among students.

Because we merged the UCLA and UCSC courses, our class had a team of five instructors (the five authors of this manuscript). In addition, and thanks to funding from HHMI, we employed five graduate teaching assistants (TA) to lead discussion sections and assist with grading (one per 20–25 students) and five undergraduate learning assistants (LA; most of whom had taken the course in 2019), for a total instructional team of 15. Although this large team was helpful for maintaining the active‐learning components of the course (e.g., Tips 8–10), it also created challenges for communication. To ensure clear communication among the instructional team, we held weekly virtual meetings during which we planned the coming week's lectures and activities, clarified each team member's roles, and tested access to necessary course materials. Once synchronous classes began, we added a Slack communication channel in which the instructional team could discuss issues privately but in real time. We used this channel, for example, to post live updates on activity progress and solve unexpected problems that arose in real time.

In addition to increased communication demands among the instructional staff, the demands of the remote learning environment led to additional needs for communication between students and instructors. However, many methods through which students normally interact with instructors were disrupted, including spontaneous meetings before and after class and in‐person office hours. We attempted to maintain opportunities for spontaneous meetings by remaining in the Zoom session after class, and each instructor held weekly remote office hours, with remote “room” links maintained in the LMS, for any student to attend.

Without face‐to‐face meetings in the classroom, opportunities for communication among students were also fewer in the remote learning setting. Although we found this to be the most challenging communication problem to solve during remote learning, we worked to maintain opportunities for students to communicate with each other through group work (see Tip 8) and by building opportunities for peer feedback into our course projects (see Tip 10).

### 2.2 Outside of class: Use a diversity of online materials and exercises that engage students with different learning styles

One major advantage of having designed a course as a flipped classroom is that most of the online, out‐of‐class material can be easily adapted for remote instruction. As we transitioned our class, we were able to incorporate our existing out‐of‐class material into our LMS. The structure of this material remained largely unchanged between remote and in‐person formats, with a few exceptions that we highlight in the three tips below. Because out‐of‐class materials can transition well between in‐person and remote formats, we encourage instructors to consider what type of materials could be reused when in‐person teaching resumes. We hope our tips will provide some inspiration.

#### 2.2.1 Tip 5. Use pre‐recorded videos, readings, and quizzes to prepare students for class

For the flipped classroom approach to be successful, students must complete assigned work outside of class in order to be prepared for in‐class activities. Following advice from the pedagogical literature (Long, Logan, & Waugh, 2016; Prud'homme‐Généreux, Schiller, Wild, & Herreid, 2017), we created short instructional videos, no longer than 15 min each, to introduce material to be discussed during class. For each class, we assigned no more than four of these videos and no more than 3–4 pages of associated readings. This relatively light and varied workload has been shown to encourage student completion of out‐of‐class assignments (Hall & DuFrene, 2016). Also prior to class, we asked students to complete a short quiz related to the video lectures and/or reading material so as to reinforce the learning process (Long et al., 2016; Szpunar, Khan, & Schacter, 2013). To motivate participation, these quizzes made up a substantial portion (15%) of the students’ final grades.

Although not only applicable to the online version of our class, we developed our materials to reflect a diversity of people and their experiences in STEM. Each member of our teaching team recorded at least one video that aligned with their expertise, and guest lecturers created videos covering materials that our instructional team did not. Students were therefore introduced both to the instructional team and provided with a broad survey of successful scientists of diverse backgrounds.

Creating course materials and lectures that are accessible to all students is critical to providing equal opportunities for all students, regardless of income, ability, disability, age, gender, or cultural and linguistic background (Colvard, Watson, & Park, 2018; Tobin & Behling, 2018). The confusion and additional stressors brought on by the unexpected transition to remote learning meant that ensuring access for all was an even bigger priority. To this end, we only used Open Educational Resources that did not require any fees or subscriptions. We also made all videos and readings accessible via a private YouTube channel, in which we set the view mode to “Unlisted—anyone with link can view.” Links to each video were provided in the LMS modules, and all videos were professionally closed‐captioned to facilitate accessibility, including for those students using public or noisy spaces for online learning. Those wishing to incorporate videos into their classes should explore options for professional editing that may be available through their universities. We note that video lectures can also be recorded using platforms such as PowerPoint or Zoom, and close‐captioned using free or low‐cost closed‐captioning software such as YouTube. Although developing video lectures and modules may seem onerous, these can be reused each year, including to transition courses to a hybrid or flipped format when in‐person instruction resumes.

#### 2.2.2 Tip 6. Give students grade‐earning options tailored to their interests, including elective and extra credit assignments

In addition to in‐class assignments (see Tip 8), we required some assignments to be completed outside of class throughout the term, with the goal to increase student exposure to a range of scientific ideas and disciplines. We created a total of 10 elective assignments from which students could choose any three to complete throughout the term, including exercises such as attending seminars, reading published literature, and participating in field excursions (see “Description of elective assignments,” Appendix S1 for all options). Given the large number of options, students could select the assignments that best aligned with their interests and with their personal situations. We added an optional fourth elective that students could choose to receive extra credit. Many of these assignments adapted well to remote learning but some assignments were field‐based and required creative solutions to adapting to a remote learning format in 2020.

Although our in‐person course had a strong field component, an unfortunate reality of the COVID‐19 pandemic was that we were not able to conduct any in‐person field trips. In 2019, we took students on day‐long and overnight field trips, where they completed field observations and collected eDNA samples. We attempted to preserve some field‐based components in 2020 using the elective assignments (see “Description of Elective assignments,” Appendix S1). For example, an elective that asked students to use the online platform iNaturalist (https://www.inaturalist.org) could be completed without moving far from home. However, other electives, such as participating in a field collection event, were not possible or safe to complete during COVID‐19 quarantine. To address this limitation, we provided a “virtual field trip” in which an eDNA collection event was live‐streamed via Zoom. Students who logged on to the transmission experienced researchers describing and recording the field environment while collecting samples along the Los Angeles River. Participating students were able to make observations and ask questions while researchers were collecting samples and other metadata, as well as experience a field collection event in real time. Students could also participate asynchronously and receive credit for the elective by watching the recording, asking questions, and providing feedback to researchers up to a week after the trip was completed.

Student feedback revealed differing appreciations of the elective assignments (Figure 3). Some students appreciated the opportunity to focus on topics of personal interest, whereas others felt that these assignments were not useful as they were not directly related to the course material. They referred to these assignments as “busy work” and did not see the growth opportunity. In the future, we will try to communicate the professional development goals of these assignments more clearly such that our students better understand their value.

#### 2.2.3 Tip 7. Engage your students through videos and essays that allow them to express their personality and aspirations

The paucity of interpersonal interactions during remote instruction may lead some students to feel isolated and disinterested, which works against our goal of engaging early undergraduates in STEM subjects. We created an online video essay assignment that allowed instructors to become more personally acquainted with students as well as increase students' personal connection with science, one of our major learning goals. We created an assignment where students explored and presented personal visions of themselves in their future careers (described below and in “Career video assignment,” Appendix S1). This type of assignment could be easily adopted to many courses in Ecology and Evolution and would be useful in remote instructional settings where meeting students in person is unlikely.

We asked students to watch five of a possible 14 testimonial videos made by scientists in academia, government, industry, journalism, and other careers. These video testimonials were created by scientists at different career stages and from diverse socioeconomic and academic backgrounds. In each video, the career scientist describes their personal motivations and path to their current position and provides advice for students wanting to pursue similar careers. After watching these videos, students were asked to record their own video or, if they preferred, to write a short essay reflecting on their proposed career path and aspirations. Through this assignment, students both considered their own career goals early in their college experience, perhaps even before declaring a major, and built personal connections to the instructional team. In their videos and essays, many students reported that their view of scientists changed positively after watching the videos and that they enjoyed the opportunity to better understand the different career paths and day‐to‐day lives of scientists in different fields. Many students expressed gratitude to be given a chance to reflect on their own goals and aspirations. In their essays and videos, students often contextualized the challenges they faced or the careers they are considering by describing their family and/or cultural background. This information helped instructors discover facets of our students otherwise difficult to identify, especially in the remote format. Such information allowed us to create a more inclusive learning community and tailor the course content to student needs and interests.

### 2.3 During class: Maintain an active‐learning environment online

In‐class time undoubtedly requires the most reworking when transitioning from in‐person to remote teaching, particularly in the flipped classroom format. That being said, the two previous sections—tips on adjusting your course before and outside of class—should set the stage for the delivery of the class material in terms of logistics, communication, and student prior knowledge. Below, we present three tips that we used to encourage and track student engagement during remote instruction and suggest project‐based assessments as alternatives to traditional examinations that may be problematic to implement when delivered remotely.

#### 2.3.1 Tip 8. Encourage student engagement during remote classes

Our 2019 course plan included activities that fostered collaborative learning (Tanner, 2013; Tanner, Chatman, & Allen, 2003) such as jigsaw activities, group discussions and presentations, software tutorials, and an Oxford‐style debate. We found that most of these activities could be adapted to a remote format using the “breakout room” function in Zoom. For each activity, we created spontaneous small breakout groups comprising 6–10 students each. One facilitator (instructor, TA, or LA) was assigned to each breakout group (facilitators often moved between more than one breakout group), and to encourage participation among students, each student was assigned a particular role in their group, such as note taker or presenter (Tanner et al., 2003). For most in‐class activities, each breakout group was asked to complete a worksheet or to work through a concept. To facilitate this effort, we created Google worksheets for each breakout group so that students could collaborate easily on a shared document and provided unique links to each groups' document at the beginning of the breakout session. When the activity ended, students returned to the main room and one team member reported back to the rest of the class. Without this reporting option, we found that students left the class without completing the breakout activity. Students saved their completed Google worksheets as word documents or PDFs and uploaded them for assessment in the LMS.

We found that activities ran smoothly and more students participated when clear and concise instructions were presented at the beginning of each activity. We noted that student engagement with breakout rooms varied depending on each student's personal situation; some were unable to use video and others unable to use either audio or video. Those students who were able to use both audio and video were often more engaged than those that chose not to or were unable to do so. Engagement also improved when the type or trajectory of the group activity was new, as students appeared to fatigue more quickly in the online format than in person, despite that the composition of the groups was different for each activity. We present several breakout room activities as Appendix S1 (see “Jigsaw activity,” “Software tutorial,” and “Oxford‐style debate”), including example worksheets and general activity structures.

While written instructions allowed students to work independently, we found value in having a facilitator present in each breakout room or moving between several breakout rooms to ensure that students understood the instructions and felt comfortable communicating with each other. Student feedback indicated that students valued the presence of facilitators in the breakout rooms and that they appreciated the small‐group settings for conversations with both peers and facilitators. For classes that may not have a large instructional team to facilitate all breakout rooms simultaneously, we also found it possible for instructors to monitor Google Worksheets in real time and move to groups that appeared to struggle as a means to track group participation and progress.

Student opinions of breakout rooms and collaborative activities were conflicting. Although many found these activities to be helpful to their learning (Figure 3) and a welcome opportunity to interact with peers, others felt that interactions were awkward and that work was not shared equally among group members. These problems are not unique to remote learning (Cohen, 1994). Measures that may address these challenges include maintaining the same breakout rooms throughout the course such that students become more familiar with each other and thus more active in discussions or creating even smaller groups, as some students felt the smaller group experiences were more valuable. We note, however, that groups with varying membership and of relatively large size provide more opportunities for students to engage with each other.

#### 2.3.2 Tip 9. Keep track of attendance and participation

Assessing student attendance and participation can be a challenge in all course settings, but is particularly challenging when teaching remotely. During the in‐person class in 2019, we used “name tents” to introduce students to each other and to us and, in subsequent classes, to track attendance. To encourage individual participation, we used activities such as think‐pair‐shares and one‐minute papers. These activities do not transition easily to remote learning, which required different approaches to both.

Many online options allow instructors to track student engagement. For example, some LMS track engagement by logging email addresses of students who join remote classes from the link within the LMS module. Because our class spanned two university campuses and two different LMS, this option was not available to us. Instead, we used Google Forms to collect real‐time student responses to questions posed during class and log attendance, and interactive participation tools in Zoom, including polls and chat, to engage students directly during the lecture.

We found Google forms to be particularly useful to track attendance. During each class, we asked four to six questions at different times during the lecture (see “Google Forms,” Appendix S1). Both the question and link to the Google Form were provided on lecture slides and in the Zoom chat box, but the questions were not included in the Google Form itself, such that the only way the students would know how to answer the question was either to be present in the synchronous lecture or by watching the recorded lecture later. Because Google Forms provide time stamping of submitted responses, we could track which students attended the synchronous sessions. Students that were unable to participate in the synchronous lecture completed the form as they watched the class recording. Asynchronous students were also asked to briefly summarize what they had learned during the class to incentivize them to watch the full lecture recording, as opposed to only finding the slides with questions. In both cases, attendance points were awarded regardless of the correctness of the answers provided. However, students' responses, which they were prompted to share via the Zoom chat feature, were used by the instructor to better understand how well students were understanding the concepts and as a real‐time motivation for topical discussions.

The participatory functions embedded in the Zoom platform were also useful to engage students in real time. We encouraged students to ask questions either using the “raise hand” function or by typing questions into the chat box and assigned monitoring and answering roles to the instructional team. Feedback from students revealed that some were uncomfortable using either of these two options. A potential solution would be to add an item to the Google Form that allows students to ask questions, although these questions will be challenging to monitor and answer in real time.

#### 2.3.3 Tip 10. Diversify assessments: focus on projects over examinations

One aspect of remote learning that instructors have struggled with is how to handle remote assessments and final examinations when transitioning to a virtual format. Examinations can be difficult to proctor online because they require substantial planning and setup, not all students have access to the appropriate technology, and there may be concerns about cheating. Rather than adapt traditional examinations for a remote setting, instructors could choose alternative assessments, which pedagogical research has shown create more authentic means for students to demonstrate what they have learned and the skills they have developed (Dikli, 2003). Alternative assessments also have the advantage that they transition well between in‐person and remote formats and thus could be used when in‐person classes resume. Below, we explain how we used our learning goals as a starting place to create three major assessments for our in‐person flipped course. These required only minor changes when transitioning to remote format.

One of our course goals is to provide opportunities for students to develop communication skills that allow participation in the culture of research, furthering professional identity as scientists (Brownell, Price, & Steinman, 2013a; Gray, Emerson, & MacKay, 2005). Learning how to communicate effectively also improves self­efficacy and promotes student learning (Brownell, Price, & Steinman, 2013b; Pelger & Nilsson, 2016). Rather than proctor high‐stakes examinations, we aimed to create assignments that would allow students to develop and practice those scientific inquiry skills that are required to design an experiment, analyze results, and communicate research findings to diverse audiences. In lieu of examinations, our students were asked to complete three major assignments, each worth a substantial portion of their grade: (a) carrying out a research project on a topic of their choice (15%); (b) writing a research proposal (25%); and (c) delivering an oral presentation on a scientific paper (10%). Unfortunately, the due dates of both the written proposal and oral presentation coincided with amplified racial unrest throughout the United States during the Spring of 2020. While racism, inclusivity, and accessibility are not new issues in universities, many of our students or their families were directly and acutely affected by these events. Given these circumstances, we chose to make the final proposal and group presentation optional assignments in 2020. We therefore focus here on describing the research project, which was required for all students and due at the midpoint of the quarter in lieu of a midterm examination.

Our research project assignment capitalized on data generated by the California Environmental DNA (CALeDNA) community science program (Meyer et al., 2019) to increase student engagement through active and inquiry‐based learning. In this structured activity, students combined community science data with open‐access bioinformatics tools (Kandlikar et al., 2018; https://gauravsk.shinyapps.io/ranacapa/). They also experienced authentic research by developing their own research questions, hypotheses, and predictions, testing them, and writing reports to communicate with both scientists and nonscientists.

The project provided opportunities for students to work individually and in teams and to get instructor and peer feedback before the final report was due. Students first individually wrote a research question that was interesting to them. Instructors created student research teams of 4–6 students based on topic similarity, and students worked for the remainder of the project in these teams. They developed their hypotheses and predictions together, while instructors and TAs interacted with the teams throughout the research project to provide feedback and guide students in creating and answering their research questions. Projects were scaffolded into multiple stepwise assignments with the goals of (a) increasing instructor feedback to promote student learning of the scientific process (Stewart‐Mailhiot, 2014), and (b) reducing the pressure of high‐stakes assignments (e.g., term papers, examinations that are typically only graded as a final product) which can impede student learning (Harland, McLean, Wass, Miller, & Sim, 2015). Within each scaffolded assignment, teams explored data, tested their hypotheses and discussed their findings, building on each other's knowledge as the assignment progressed. We encouraged students to meet outside of class and also provided class time for teams to collaborate. Part of their grade on the assignment was to provide peer feedback during class. While the bulk of the data exploration and analysis was completed as a team, final reports were written by each individual student. Thus, students could build on their interactions with teammates, but were ultimately responsible for their individual grade on the assignment. The midterm research project was more popular among students in 2020 than 2019, suggesting that students appreciated the opportunity to work in groups and participate in authentic research despite the challenges associated with remote group work. However, it is also possible that the research project assignment was better implemented by instructors in the second year.

## 3 CONCLUSION

The COVID‐19 pandemic is a global health crisis that continues to require educational institutions to find creative ways to restructure course delivery while maintaining quality instruction and equitable outcomes for students. Above, we presented ten strategies that we found most valuable when restructuring our in‐person flipped classroom for remote instruction. These ten tips focus on strategies to develop an online learning community that is both fair and inclusive and that empowers students to acquire conceptual knowledge and develop scientific inquiry and literacy skills. In implementing these strategies, we hope to have developed spaces where students can gain new understanding of taught concepts and reflect on personal connections with biology and their desired career path (Figure 1).

Despite the difficulties associated with remote learning, we found from student interactions and feedback that most students approached the remote learning experiment with a positive attitude. Not only did survey data indicate little difference in student's learning of concepts and skills between in‐person and remote teaching, we also were pleasantly surprised at the level of student engagement both inside and outside of the remote classroom. For example, student participation and attendance remained high throughout the 2020 term.

Finally, we acknowledge that our recommendations will not fully bridge the learning disparities that will inevitably continue to arise as remote learning remains a reality throughout colleges and universities. Institutions and instructors must remain mindful of other situations that perpetuate those disparities that are still widely present in our universities. Ours, like many courses, faced additional challenges toward the end of the spring quarter with the widespread uprisings in response to long‐standing systemic racial injustices and following the murders of George Floyd, Ahmaud Arbury, Breonna Taylor, and others. While our course objectives and adaptations to remote learning intended to address issues of student inclusivity and learning equity, these events combined with the ongoing pandemic further highlighted the disparities among our students. In particular, ensuring access to technology for each student and financial support in situations of hardship are essential in delivering an online active‐learning course. In the future, we will strive to ensure these resources are available to our students even during in‐person instruction. As institutions continue to adapt their courses to the changing educational landscape, we hope that instructors across STEM fields can draw inspiration from our experience and determine practical solutions for ensuring student inclusion and scientific curiosity during online teaching.

## 4 GLOSSARY

*Active learning*: An educational method in which students participate or interact with the learning process, as opposed to the more traditional passive method of learning in which they watch their instructor lecture without interaction. Active learning may incorporate a variety of activities that promote engagement and critical thinking such as think‐pair‐shares, group discussions, jigsaw activities, and research projects.

*Asynchronous online learning*: Learning that occurs when students do not participate in class at the same time as it is being taught by instructors. Instead, they watch a recording of class and complete any activities in their own time. This learning option is valuable for students in different time zones or who have caring or work responsibilities during scheduled class time.

*Bioblitz*: A community science event during which scientists, naturalists, and volunteers (including families and students) meet to survey biodiversity in a given area and period of time. The CALeDNA science initiative (https://ucedna.com/) organizes bioblitzes during which participants are given eDNA sampling kits to collect samples and contribute to biodiversity databases.

*Breakout room*: An online space where smaller groups of students and instructors can meet separately from the larger classroom. A specific number and/or group of students per room can be set in advance or students can be assigned randomly.

*Environmental DNA (eDNA)*: DNA collected directly from environmental samples, originating from shed material such as hair, feces, leaves, or microbes that can be found in a variety of environments (e.g., sediment, water, snow, soil). eDNA methods provide a noninvasive way to survey organisms that are likely present in an ecosystem.

*Flipped classroom*: Students use resources such as pre‐recorded videos or readings outside of class time in preparation for class, which typically replace a traditional lecture. The time that is usually spent lecturing during class can be replaced with active‐learning techniques.

*In‐person learning*: Instructors and students meet face to face for class rather than online.

*Inclusive teaching*: Teaching approaches that consider the diverse backgrounds and needs of students so that meaning and accessible learning are available to all students regardless of income, ability, disability, age, gender, or cultural and linguistic background.

*Jigsaw activity*: A cooperative learning method that allows students to learn through collaboration and peer‐teaching. Students are split into small expert groups in which they focus on a topic; new groups are then formed with experts from all the different topics. Each expert has to explain their findings to the other students working on different topics.

*Learning assistant (LA) or undergraduate teaching assistant*: Typically, an undergraduate student who has previously taken the class and helps deliver the class materials with the rest of the instruction team.

*Learning community*: A group of instructors and students who share common academic goals and work on classwork collaboratively. Learning communities promote interactions between instructors and students, coherence within the curriculum and focus on learning outcomes.

*Learning‐focused syllabus*: A syllabus that focuses on the students, as opposed to the content; it emphasizes what the student will learn and how the instructors will deliver the course material and support the students in achieving the learning goals. It clearly describes the questions asked in the course, highlights connections between course themes and activities, and explains how students can succeed in the course.

*Learning management system (LMS)*: An online platform where course material is made available for instructors and students. Course material can include videos, readings, assignments, examinations, and quizzes. LMS can also be used to make announcements, message students, and grade student work.

*Name tent*: A folded piece of paper or cardboard where a student writes their name and other information (i.e., preferred pronouns, major, interesting fact about themselves) and which is displayed on their desk during class. Building the name tents can be used as an ice‐breaker activity during the first class and collected at the end of each class to record attendance.

*One‐minute paper*: A short written reflection by students on a topic proposed by instructors during active learning. This activity is typically followed by a full class discussion on the topic.

*Oxford‐style debate*: A debate around one motion from two opposing perspectives. It is divided into three sections: opening remarks, questions from the audience, closing arguments. Each side—for and against the motion—takes turns debating each section. Audience members vote on the motion before and after the debate and the voting breakdown is shared at the end of the debate to see if any members’ view was swayed by the arguments presented.

*Synchronous online learning*: Learning format where students attend and participate in class at the same time as it is being delivered by the instructors (i.e., as it is live‐streamed on the videoconference platform).

*Think‐Pair‐Share (TPS)*: Collaborative learning strategy where students (a) think individually about a topic, (b) discuss idea with a few classmates, and then (c) participate in a whole‐class discussion. TPS allow students time to think before participating and give them the opportunity to make connections with class material and identify misconceptions before the topic is discussed in larger groups.

## CONFLICT OF INTEREST

None declared.

## AUTHOR CONTRIBUTION

**Ana Elisa Garcia‐Vedrenne:** Conceptualization (equal); Data curation (equal); Formal analysis (equal); Writing‐original draft (equal); Writing‐review & editing (equal). **Chloe Orland:** Conceptualization (equal); Data curation (equal); Formal analysis (equal); Writing‐original draft (equal); Writing‐review & editing (equal). **Kimberly M. Ballare:** Conceptualization (equal); Data curation (equal); Formal analysis (equal); Writing‐original draft (equal); Writing‐review & editing (equal). **Beth Shapiro:** Funding acquisition (equal); Supervision (equal); Writing‐review & editing (equal). **Robert K. Wayne:** Funding acquisition (equal); Supervision (equal); Writing‐review & editing (equal).

## Supporting information

Appendix S1

## Data Availability

Survey results and class materials can be found the Appendix S1 for this manuscript.
