# Supplementary material for: Ten strategies for a successful transition to remote learning: Lessons learned with a flipped course
Source: Ecol Evol. 2020 Oct 16;10(22):12620–34. doi: 10.1002/ece3.6760 (PMC7679550; doi:10.1002/ece3.6760)

**Supporting Information for:**

**Ten strategies for a successful transition to remote learning: lessons learned with a flipped course**

Ana E. Garcia-Vedrenne^1^*^⧫^, Chloe Orland^2^*, Kimberly M. Ballare^2^*, Beth Shapiro^2,3^, Robert K. Wayne^1^

^1^ Department of Ecology and Evolutionary Biology, University of California Los Angeles, Los Angeles, CA 90095 USA

^2^ Department of Ecology and Evolutionary Biology, University of California Santa Cruz, Santa Cruz, CA 95064 USA

^3^ Howard Hughes Medical Institute, University of California Santa Cruz, Santa Cruz, CA 95064 USA

*These authors contributed equally to this work

^⧫^ Corresponding Author, Contact information: [garciavedrenne@g.ucla.edu](mailto:garciavedrenne@g.ucla.edu)

[Table of Contents](#_7v3sod55cqis)

[Figure S1: Student self-assessed level of knowledge about course topics](#_a9t1dkkapeyr) Page 2

[Figure S2: Student self-assessed ability to perform inquiry skills](#_xp17nnnqvqv7) Page 3

[Access to technology questionnaire](#_uf92zcnuigl5) Page 4

[Syllabus (2020)](#_akzpe7w9yjnw) Page 5

[COVID19 Exceptional circumstances](#_gjdgxs)

[Syllabus Table of Contents](#_30j0zll)

[Course Description](#_1fob9te)

[Learning Outcomes for this Course](#_3znysh7)

[Course Materials](#_3dy6vkm)

[How to Succeed in this Course](#_1t3h5sf)

[Learning Community Expectations](#_4d34og8)

[How Your Learning Will Be Assessed (Grading Policy)](#_2s8eyo1)

[Course Schedule](#_u3nfvkw7sd78)

[Description of Elective assignments](#_o27y929jq9tb) Page 15

[Career Video Assignment](#_8ftjya6x8um8) Page 16

[Examples of collaborative worksheets](#_godtltgtwqe3) Page 17

[Jigsaw activity with scientific articles](#_bjynxfw8bdac) Page 17

[Software tutorial](#_o5limm1q0jel) Page 20

[Oxford-Style debate](#_kui2izv2kogr) Page 27

[Google Form](#_y76wpyus9uqx) Page 31


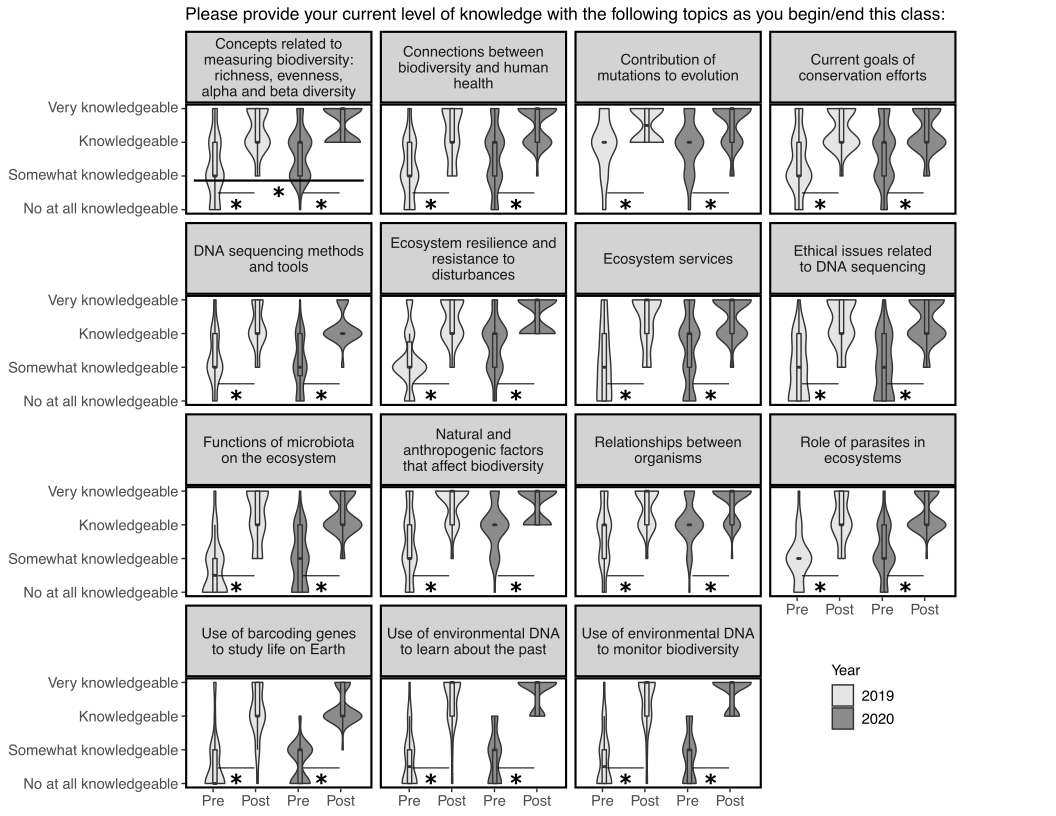


Figure S1: Student self-assessed level of knowledge about course topics before the course started (pre) and at the end of the course (post) for the in-person 2019 course (n = 30) and the remote 2020 course (n = 50). The survey provided further clarification as to the knowledge categories as follows: Not at all knowledgeable (i.e. I am unfamiliar with the topic); Somewhat knowledgeable (i.e. I have heard of the topic but could not readily explain it to someone); Knowledgeable (i.e. I have heard of the topic and could readily explain what it means to someone); Very knowledgeable (i.e. I understand current research on the topic & could teach it to a peer). The increase in perceived knowledge of the topics was significant as indicated by paired T-tests (p<0.05) for all topics in both 2019 and 2020 (denoted by asterisks (*)). Perceived increase in knowledge did not significantly vary between 2019 and 2020, except for the first topic: Concepts related to measuring biodiversity: richness, evenness, alpha and beta diversity (t(48.2)= -2.57; p= 0.0133, denoted by an asterisk (*)).


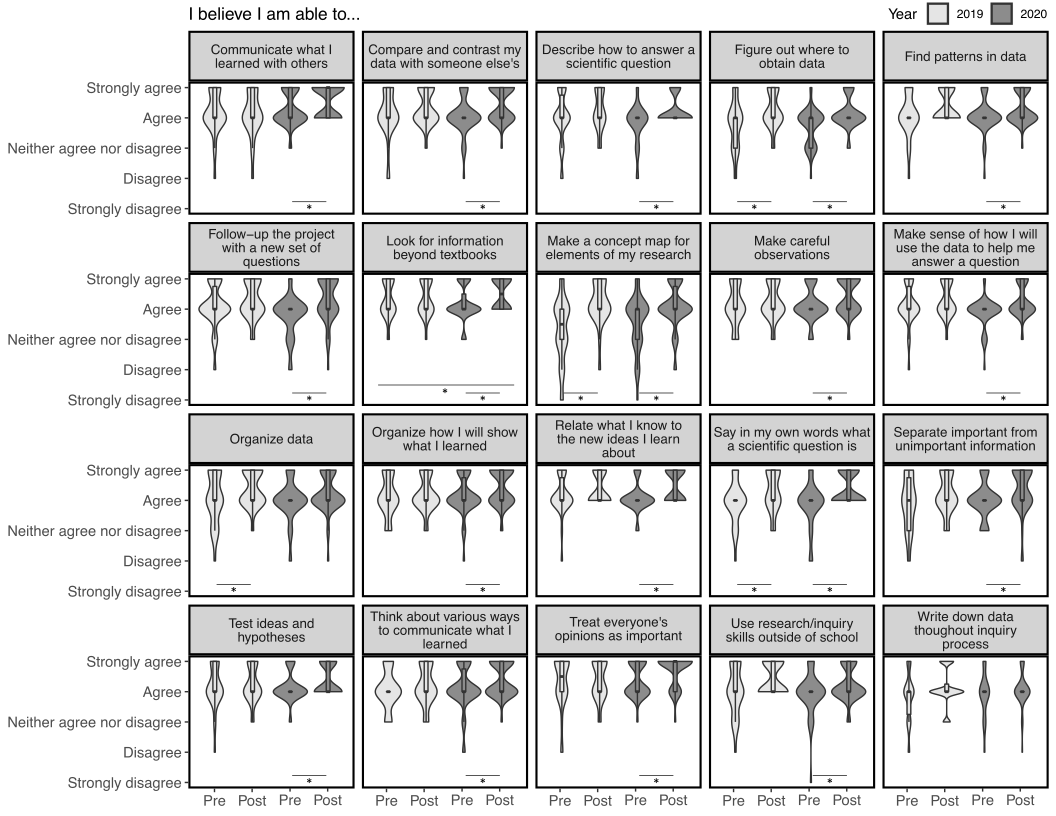


Figure S2. Student self-assessed ability to perform inquiry skills at the beginning of the course (pre) and at the end of the course (post) for the in-person 2019 course (n = 30) and the remote 2020 course (n = 50). Asterisks (*) indicate skills where the increase in perceived ability is significant (p<0.05) before and after the course. Perceived increase in knowledge did significantly vary between 2019 and 2020, except for: Look for information beyond textbooks (t(56.3) 2.44; p= 0.0176).

## **Access to technology questionnaire**

Students were sent this questionnaire as a Google Form via email prior to the start of the 2020 Spring quarter.

- What time zone will you be in for the Spring quarter?
- Do you have digital access for everyday use?
- What platforms do you use to access our CCLE/ Canvas page? (laptop, tablet, smartphone, other)
- Is Wifi everyday accessibility realistic for you?
- Do you have daily access to a webcam or microphone?
- Are you familiar with group chat/lecture services like Zoom?
- Do you foresee being able to commit to attending lecture and discussion sections?
- Based on your response above, please briefly explain your anticipated challenges with access?
- Is there anything else you would like us to know?

##

## **Syllabus (2020)**

**Biodiversity in the Age of Humans**

**Spring 2020**

**Class Hours:** Tuesday/Thursday 3:30-4:45 PM PST

**Classroom:** Zoom Classroom, link posted on Canvas

**Discussion Sections:** Zoom Classrooms, links posted on Canvas

*please attend your registered section*

**UCSC Instructors (personal zoom links in parentheses):**

*Redacted for publication*

### COVID19 Exceptional circumstances

As you all know, we are currently facing an exceptional situation with COVID19. This class was designed to be an active learning class with a lot of activities during class including group work, think-pair-shares, drawing on the whiteboard, presentations, and other activities that were designed for an in-person course.

As with all other classes this quarter, we are required to modify our course content for remote teaching at very short notice. Our aim is to keep this class as active as possible though. To do so, we will need everyone to cooperate by getting involved during the synchronous sessions (e.g. by asking/answering questions through the chat rooms or by raising your hand, by participating during group breakout sessions, contributing to group work, etc.). We understand that this may be difficult to adjust to at first, but we hope that together we can keep this class engaging and interesting.

If you anticipate any challenges regarding your ability to connect synchronously, we ask that you reach out to us. We will keep this information confidential and will discuss ways to accommodate for your individual situation. **Participation in this class should not threaten stay-at-home orders or social distancing guidelines.**  Thank you all for your cooperation and let’s support each other through these difficult times as much as possible!

This course will be taught concurrently with students and instructors from UCLA. We hope you enjoy this course as much as we’ve enjoyed preparing it.

To try to make everything go as smoothly as possible, your instructors will be available on **Monday March 30th (9am-5pm PST)**, the day before the first class, to answer any logistical questions about the online course. We will provide zoom links on Canvas. Please reach out then!

## **Syllabus (2020), cont’d**

### Syllabus Table of Contents

**Syllabus Table of Contents**………………………………………………….1

**Course Description**…………………………………………………………...2

**Learning Outcomes for this Course**……………………………………….2

**Course Materials**.……………………………………………………………..3

**How to Succeed in this Course**.…………………………………………...3

**Learning Community Expectations**……………………………………….5

**How Your Learning Will Be Assessed (Grading Policy)**……………....5

### Course Description

The influence that human activity has had on the environment will leave its legacy for millennia. Just a few of the issues at hand include loss of habitats, overharvesting, poaching, invasive species, endangered species, and climate change. How can we better understand how humans have affected the earth and its inhabitants, and what can we do to address these problems? Environmental DNA (eDNA) – DNA shed by all organisms into the environment – provides a new approach to study earth’s biodiversity and also has applications in forensics, medicine, anthropology, agriculture, conservation, and many other fields. Because eDNA can be easily collected from water and soil samples, we can study changes in distribution and abundance of animals, plants, fungi, and microbes. In this class, you will have the opportunity to collect your own eDNA samples, make field observations, and consider how to use eDNA to solve an environmental issue. This course will teach you how to use the scientific method, how to ask and answer questions about eDNA, analyze literature, and develop professional skills applicable to any major or career. A series of videos, interactive worksheets, and short lectures outside of class will set the baseline knowledge for our problem solving and applied learning in the classroom. You will complete the course with an appreciation of modern scientific methods and research, and a skill set that will serve you well regardless of where you go next. All majors are welcome; no background in life sciences is required.

### Learning Outcomes for this Course

**Learning Outcomes:**

By the end of the course, students will be able to:

- Define environmental DNA (eDNA) and explain how this method can further STEM research and knowledge of biodiversity
- Outline the steps of the scientific method and the application of this process to biodiversity studies
- Read, interpret, and create graphs and data

## **Syllabus (2020), cont’d**

- Synthesize primary literature on eDNA studies
- Argue why biodiversity is important for human and ecosystem health
- Design an experiment using eDNA to ask a scientific question relevant to a STEM field of interest to them
- Teach fellow students about current biodiversity research through in-class student presentations
- Develop an individual research proposal regarding biodiversity

### Course Materials

Course website: Canvas

Zoom Classroom: TBD (please include your first, last name and preferred pronouns on your username)

Course readings and videos will be posted to the course website. Zoom recordings of each lecture will also be posted.

CALeDNA website:<http://www.ucedna.com/>

Smartphone, ipad, or laptop. You will be using computers to collaborate, engage in technology-based exercises, and to reflect on your learning. If you do not have a portable device, please **contact us ASAP for guidance and assistance. This will be particularly important for us to know in the current context of COVID-19 and as we develop the course online.**

How to Succeed in this Course

There are many ways for you to show what and how you are learning: through your effort, interaction, application of science to the real world, and performance on projects.

This class has been structured to help all students get the support and guidance needed to succeed. This may feel like A LOT of work, but it is intended to relieve anxiety while encouraging practices that help you learn the material at a reasonable pace and creatively.

This is a 5-credit class, which means you are expected to work an average of **15 hours per week**. Below is a list of activities you will be asked to complete throughout the quarter.

| **Action** | **Description** |
| --- | --- |
| **Ask questions!** | You can ask questions in: 1) class; 2) office hours; 3) discussion section; 4) an online discussion forum; and 5) online via the chat room. |
| **Watch online videos** | Videos will help you become familiar with the concepts covered throughout the week and prepare you for the in class and online activities where we will further explore the weekly topics. |
| **Participate in lectures** | Rather than passively listening and writing, this is a class very much about doing and thinking. In lecture, we will have multiple activities which will encourage you to think and apply your knowledge. |
| **Participate in discussion sections** | You will learn and practice skills that will help you perform well both in this class and throughout your whole undergraduate experience.  The discussion section will consist of a variety of topics, including:   1. Reinforcement of lecture topics 2. Time to work and get feedback on group or individual projects 3. Professional development skills: oral presentations, resume building, writing skills |
| **Complete assignments** | Use them to develop and practice your new skills and to identify other questions to ask.  Don’t forget to work with others: post your question on the discussion forum, ask your TA or peer tutor, and/or come to office hours to get help when you are stuck. |
| **Study for quizzes** | Quizzes are designed to test your understanding of the material. If you didn’t understand a quiz question, ask the instructor/TA/peer tutor for help please! Do it now, before we keep building on this material and it gets more confusing. |
| **Analyze real eDNA data** | For your midterm project, you get to work with eDNA data that no one had looked at before! Explore the CALeDNA website and use specialized software to answer questions about a topic of your interest. |
| **Teach peers about eDNA research** | Being able to communicate effectively is a skill that will come in handy no matter which career path you choose. Teach fellow students about current biodiversity research through in-class student presentations. |
| **Write a research proposal** | Now you get a chance to design your own research project. Which topics in the class were the most interesting to you? Are there any unresolved issues in that field that you would like to investigate? |
| **Submit personal reflection** | Record a short interview about yourself and your career path. Who are you? How did you achieve the position you are in now? What makes you most excited about your career? What are your goals for the future? What are some obstacles you’ve encountered along the way and how have you overcome them? Any advice for someone who might want to follow in your footsteps? |
| **Complete 3 elective assignments** | In addition to the required assignments, we ask that you complete 3 elective assignments throughout the course. These give you an opportunity to tailor your learning to your personal interests. Details for each of these elective assignments will be posted on Canvas. |

### Learning Community Expectations

This course is designed for us to be a **learning community**, where all of us have individual contributions and questions that can enhance our learning experience. With that in mind, we have strived to make this course be an engaging and a welcoming environment that inspires critical thinking, creativity, and continual feedback. We have developed a variety of activities and have a strong support system of Instructors and Teaching Assistants to help answer any questions or concerns regarding difficulties or improvements for this class. You can contact anyone from the team in class, via email, and during office hours. If these times are not convenient, you can also schedule another time to meet.

In this class, we will work to promote an anti-discriminatory environment where everyone feels safe and welcome. Discrimination can be direct or indirect and can take place at both the institutional and personal levels. Discrimination of any kind, such as harassment, bullying or discrimination is unacceptable and we are committed to providing equal opportunity. The success of this policy relies on the support and understanding of everyone in this class. We all have a responsibility not to be offensive to each other, or to participate in, or condone harassment or discrimination of any kind. Any acts of discrimination are taken seriously, will be fully investigated, and may have dire consequences.

**Late Attendance/Absence Policy**: **Attendance at all lectures and your scheduled discussion section is required.** We will be taking attendance at each class and this will be factored into your participation grade (but see below).

**Academic Honesty**: Any suspicion of academic dishonesty (i.e. cheating or plagiarism) will be taken seriously and dealt with according to university policy. <https://ue.ucsc.edu/academic-misconduct.html>

## **Syllabus (2020), cont’d**

### How Your Learning Will Be Assessed (Grading Policy)

**Grading policy:** If you ever feel that an assignment or specific question is unfair or confusing please come and speak with an instructor or your TA (ideally before it is due or during the assessment) so that we can address this concern as soon as possible. We are committed to making sure the assessment of your learning is comprehensive, fair, and incorporates best practices from education research on assessment design and inclusive practices. Grades will be regularly updated on the course website.

Course announcements, schedule changes, feedback on your work, and grades will be primarily shared via Canvas. **You are responsible for keeping up-to-date with Canvas announcements and making sure you receive email notifications (check your notification settings on Canvas).**

**In-Class Participation: 15%**

Option A: Participation in class and online will be based on short questions that will be answered individually using Google Forms, polling technology, posting questions online, contributing to class discussions, and completing individual and group tasks assigned in class. We highly encourage everyone to attend and participate in synchronous sessions. It will be easier to complete the assignment with teamwork!

Option B: We understand that given current conditions it might be impossible for you to attend all sessions (due to sickness, having to care for someone else, no access to reliable internet, etc). To relieve stress due to this, we will not penalize those unable to join synchronously as long as you complete the work assigned during your absence and submit by the designated deadline. All the lectures will be recorded and you will be able to watch them in your own time. If you cannot attend synchronously, simply watch the recording and submit the corresponding Google Form. There is one question at the end that need only be answered by students selecting Option B. You have one week after the class to submit all work related to that lecture.

We recommend that you participate (Option A) to get the most out of the class, however if you cannot participate in synchronous sessions, you can always take advantage of Option B. You may choose Option B either for the entire term or as needed on a class-to-class basis.

**Assignments: 20%**

Assignments can range anywhere from quizzes, worksheets, group activities or homework based on videos and readings. Quizzes must be completed before the start of the class. The two quizzes in which you score the lowest will be dropped from your grade. Regardless of whether you choose Option A or Option B, in-class assignments are due one week after the class. Only on time and completed assignments are considered for grading.

In addition to the required assignments, **you are required to complete 3 of the available elective assignments**. Details about what is required for each activity are available on the course Canvas site.

## **Syllabus (2020), cont’d**

**Midterm project: 15%**

You will develop a research question and hypothesis about eDNA and analyze real data to answer your question. You will write a brief report that includes your question, hypothesis, results, and conclusions.

**In-class group presentation: 10%**

In groups of 4-5 team members, you will record a presentation on using eDNA to solve a problem in any of the topics covered throughout class. Each team member is required to speak and produce one slide that they will present. The presentation is to be between 8-10 minutes. You will be graded based on the rubric posted on the course website. Guidance on giving oral presentations will be given by your instructors. You will also be asked to watch and peer evaluate the presentations from two other teams.

**Final Paper: Individual eDNA research Proposal: 25%**

Detailed instructions will be provided in class and/or posted on the course website.

**Discussion section assignments: 15%**

Detailed instructions will be provided in class and/or posted on the course website.

| Letter Grade | Percentage |
| --- | --- |
| A+ | 99-100% |
| A | 93%-98.9% |
| A- | 90%-92.9% |
| B+ | 87%-89.9% |
| B | 83%-86.9% |
| B- | 80%-82.9% |
| C+ | 77%-79.9% |
| C | 73%-76.9% |
| C- | 70%-72.9% |
| D | 60%-69.9% |
| F | 0%-59% |

### Course Schedule

***This is a tentative schedule and subject to change, with schedule adjustments posted on the course website.**

## **Syllabus (2020), cont’d**

**Additional course readings and videos will be posted to the course website website.**

| **Date** | **Topic** | **Complete Before Class** |
| --- | --- | --- |
| Week 1:  March 31 | Biodiversity & DNA | Video: Introduction to course  Register for Zoom |
| Week 1:  April 2 | Biodiversity & DNA | Videos:  Introduction to biodiversity and DNA  Introduction to environmental DNA  Quiz |
| Week 2:  April 7 | eDNA & Ecology | Videos:  Organisms to ecosystems  How wolves change rivers  Reading: Estes et al. (2011)  Research on assigned species  Quiz |
| Week 2:  April 9 | eDNA & Earth’s Microbiome | Video: Microbiome  Quiz |
| Week 3:  April 14 | eDNA & Evolution | Videos:  Unity and diversity of life  Adaptation  Tree diagrams  Shared functions, shared genes  Common Ancestry  Quiz |
| Week 3:  April 16 | eDNA & Evolution | Videos:  What is a species?  DNA as a method of exploring biodiversity  DNA metabarcoding  Quiz  Elective assignment #1 due |
| Week 4:  April 21 | eDNA: extraction to bioinformatic analysis | Videos:  From soil to sequence: Part I  Quiz  Personal career path video/essay due |
| Week 4:  April 23 | eDNA: extraction to bioinformatic analysis | Videos:  From sequence to analysis: Part II  Introduction to Ranacapa  Reading: Curd et al. (2019) or Kandlikar et al. (2018)  Quiz |
| Week 5:  April 28 | Research tools: Questions and hypotheses | Videos:  Sanger sequencing  Next generation sequencing  Single molecule sequencing  Research question for midterm project |
| Week 5:  April 30 | Research tools: Data analysis | Progress on midterm project |
| Week 6:  May 5 | eDNA & Conservation Biology | Videos:  Human impacts on biodiversity  Ecosystem services  Quiz  Midterm project due  Submit paper for group presentation (discussion) |
| Week 6:  May 7 | eDNA & Conservation Biology | Video: Shifting Baselines  Quiz |
| Week 7:  May 12 | eDNA & Paleobiology | Come ready to debate!  Video: Introduction to paleoecology  Quiz  Elective assignment #2 due  First draft of final proposal (discussion) |
| Week 7:  May 14 | eDNA & Climate Change | Reading: Assigned papers  Videos:  Sequencing ancient DNA  Passenger pigeon  Quiz |
| Week 8:  May 19 | eDNA and Human Health | Video: Role of parasites in ecosystems  Quiz |
| Week 8:  May 21 | eDNA and Human Health | Reading: Huver et al. (2015)  Quiz |
| Week 9:  May 26 | eDNA and Society | Video: Ethics and eDNA research  Quiz  Practice talk on group presentations (discussion)  Second draft of final proposal |
| Week 9:  May 28 | eDNA and Society | Career pathways workshop |
| Week 10:  June 2 | Group Presentations | Group presentations  Elective assignment #3 due |
| Week 10:  June 4 | Group Presentations | Group presentations |

Finals week: Final proposal due on Wednesday, June 10

##

## **Description of Elective assignments**

| **Learning Goal Category** | **Elective assignment** | **Description** |
| --- | --- | --- |
| Peer or Self-Instruction | Contribute to a Class Glossary | Student defines a term used in a video, in-class lecture and/or activity that was unclear to the student. Glossary is a collaborative document made available to all students as a class resource. |
|  | eDNA research database | Student posts the title of a research article on eDNA and a short summary of the take-home messages. This is also a collaborative document made available to all students. |
|  | Explain a class topic | Student makes a short video presentation on a class topic of their choice. |
| Professional Development | Submit CV or resume | Student will receive detailed feedback from at least one instructor or TA on the structure and content of their CV. |
|  | Interview professor or professional | Student records a video or writes the interview of a scientist discussing their career path. |
| Outside Expertise | Museum Workshop | Student discusses the importance of museums in science with UCSC Natural History Museum Assistant Director (synchronous on-line workshop). Remote teaching in 2020 meant this elective was available across campus. |
|  | Attend in-person or online seminar | Student attends a departmental or other research seminar broadly related to class topics and submits a short summary. |
| Field Work and Citizen Science | Record iNaturalist observations | Student joins the course project on iNaturalist and submits at least 20 observations. |
|  | Attend CALeDNA bioblitz | In 2019 (in person): Student participates in a CALeDNA bioblitz.  In 2020 (remote): Student attends a virtual field trip (See Tip 7) |
|  | Provide feedback on CALeDNA website | Student submits a short essay giving constructive feedback on the CALeDNA website (www.ucedna.com). |
|  | Field Journal | Student submits a descriptive narrative of natural observations, including drawings or photographs and detailed descriptions of three species observed. |

## **Career Video Assignment**

Watch the videos in the “Career interview videos” module on Canvas or Moodle. We recommend that you watch at least 5 videos. Although you may be tempted to pick the ones that seem the most relevant to your interests, we suggest that you also watch videos that may not appear as such, as each of the interviewees’ stories are unique and may inspire you in ways you do not expect.

The assignment is to make your own video, based on the videos you will have watched. You should try to answer some of the following questions:

- Who are you?*
- When and how did you first discover your interest in science?*
- What are your goals for the future?*
- What makes you most excited about a potential career as a scientist?*
- What are some obstacles you’ve encountered so far? How did you overcome them, and was it worth it?*
- Has your original perception of a scientist’s job changed throughout this course?

* Question also answered by professional scientists in their video

## **Examples of collaborative worksheets**

### *Jigsaw activity with scientific articles*

**EEB108/BIOE19**

**Week 7-2 Activity:**

**Using eDNA to understand the past**

For this activity, each group has been assigned a different paper to read and analyze. You must have **read the full paper** before coming to class. The activity will be split up into two parts. During the first part, groups will prepare an oral presentation (with no slides) that you will present to another group in the second part of the activity. Your presentation should last about 8-10 minutes.

**PART I (20 min):**

You will be put in a breakout room with students who were assigned the same paper to read. Your task during this first part is to answer the questions at the bottom of this worksheet in preparation for the presentation you will have to give in the second part. You also have to prepare your presentation (see Part II for more information on what you have to do). Complete this table and assign the presenters (pick two students from your group: one should focus on the general introduction of the paper and one should focus on explaining a specific figure).

| **Student Name** | **UCSC or UCLA** | **Email Address** | **Group Role**  **(researcher or presenter)** |
| --- | --- | --- | --- |
|  |  |  |  |
|  |  |  |  |
|  |  |  |  |
|  |  |  |  |
|  |  |  |  |

**Assignment: Submit this worksheet individually on CCLE/Canvas (i.e. answer the 6 questions specific to your paper). This is the only assignment you have to submit today.**

**ASYNCHRONOUS STUDENTS: You just need to submit this worksheet (Part 1, just the questions, not the presentation) and do not need to do Part 2.**

### *Jigsaw activity with scientific articles, cont’d*

**PART II (20 min):**

We will merge your breakout room with another group’s - the other group was assigned a different paper. In your presentation, you should introduce the paper so people who have not read it can understand the aims of the study. You should then describe one of the figures from the paper (i.e. the one at the end of this worksheet), and answer assigned questions about the figure. You will not present slides, however, feel free to prepare notes during Part I that you can use during the presentation. An instructor will be present throughout both groups’ presentations.

Make sure to cover the points below in your presentation:

**1) General introduction about the paper**

· What is the title of the paper?

· What was the aim of the study?

· Briefly describe the data used: where were the samples collected and what were

they?

**2) Explain the main figure from the paper**

· We will provide a slide of the figure as a visual aid during your presentation but you shouldn’t prepare any slides.

· In your presentation, you are required to answer:

o What is the aim of this figure? What does this figure generally show?

o Explain what is represented on the axes.

o What controls (if any) were used in the experiment?

o Are there experimental limitations to the results that are being presented in this figure?

o The six specific questions assigned to your group (see below).

### *Jigsaw activity with scientific articles, cont’d*

**Group 1**

**Ancient DNA Chronology within Sediment Deposits: Are Paleobiological Reconstructions Possible and Is DNA Leaching a Factor?**

Haile et al. (2007). Molecular Biology and Evolution, vol: 24 (4) pp: 982-989

Present on Figure 2


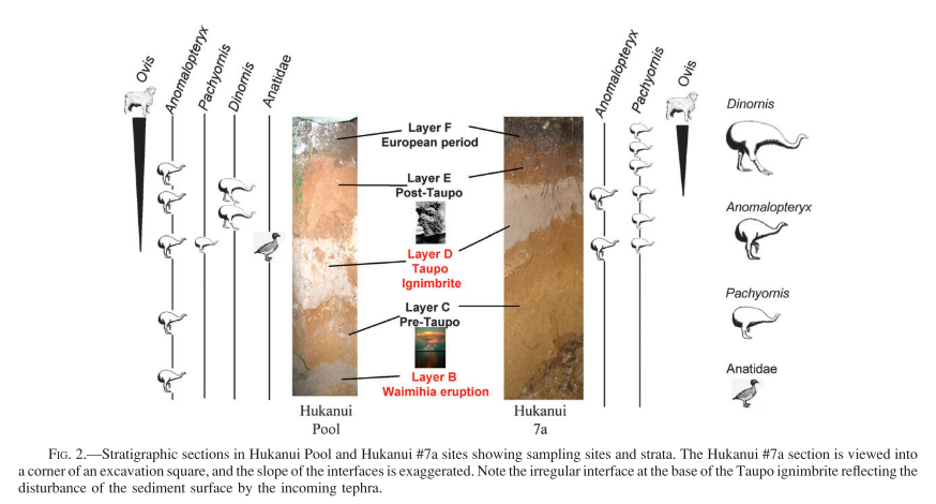


1. How do the sediment layers B-F differ from one another and what can we learn from these different layers?

2. Why was it interesting for the researchers to study these two sites specifically? How do these sites differ and what do they have in common?

3. Was the moa DNA found where researchers were expecting it? Describe why or why not.

4. Was the sheep DNA found where researchers were expecting it? Describe why or why not.

5. Why did researchers also look for Anatidae DNA?

6. What does this figure reveal about the limitations of using eDNA to reconstruct the presence/absence of species in the past?

### *Software tutorial*

Name: ______________________________ Discussion section: __________________

Instructions

1. You will be assigned a breakout room number with other group members and at least 1 instructor and/or TA.
2. You have until the end of the class to work through the tutorial. You will work through individually (but don’t hesitate to ask your group or instructor if you’re confused or need help). **Everyone will need to have several windows open on your computer at once to complete the activity, try not to get frustrated with this!**
3. Read all information carefully and follow each step in the following document to complete the exercise (labelled as **STEPS**). Complete answers to questions as indicated (Labelled as **QUESTIONS**). You will find important information to help you complete the steps and answer the questions in diagrams throughout this document (labelled as **FIGURES**).
4. Raise your hand (on zoom) if you have any questions and someone from the instructional team will come as soon as possible.
5. Save as a Word Document or PDF (File → Download) and submit to the assignment tab in CCLE/ Canvas.
6. Once you complete the tutorial and feel comfortable using Ranacapa, you are welcome to sign off. We will not be regrouping today.

Introduction: Using Ranacapa to explore metabarcoding data

Last week we talked about DNA barcoding (the use of a standardized DNA sequence as a means to identify new species, identify unknown samples, and compare relatedness and evolution among different species).

This week we will focus on understanding and interpreting **metabarcoding data**. Metabarcoding is the large-scale taxonomic identification of complex environmental samples via analysis of DNA sequences for short regions of one or a few genes (called DNA barcodes). Metabarcoding relies on high-throughput DNA sequencing (HTS) technologies, which yield millions of DNA sequences in parallel and allow large-scale analysis of environmental samples.

Aim

Today, your job is to:

- Use the bioinformatics tool Ranacapa to explore biodiversity using results from environmental DNA (eDNA) analyses.
- Analyze and interpret metabarcoding results.
- Manipulate ASV tables and metadata files effectively to retain relevant data only.

### *Software tutorial, cont’d*

Instructions

**EP 1.** Download the files in the Midterm project folder on CCLE/Canvas

**STEP 2.** Open the following website: <https://gauravsk.shinyapps.io/ranacapa/>

**STEP 3.** Open the **DATA IMPORT** tab. Because you will be using data generated specifically for this class, you should select “Custom” dataset as shown in **Figure 1**. You should now see the option of uploading two files.


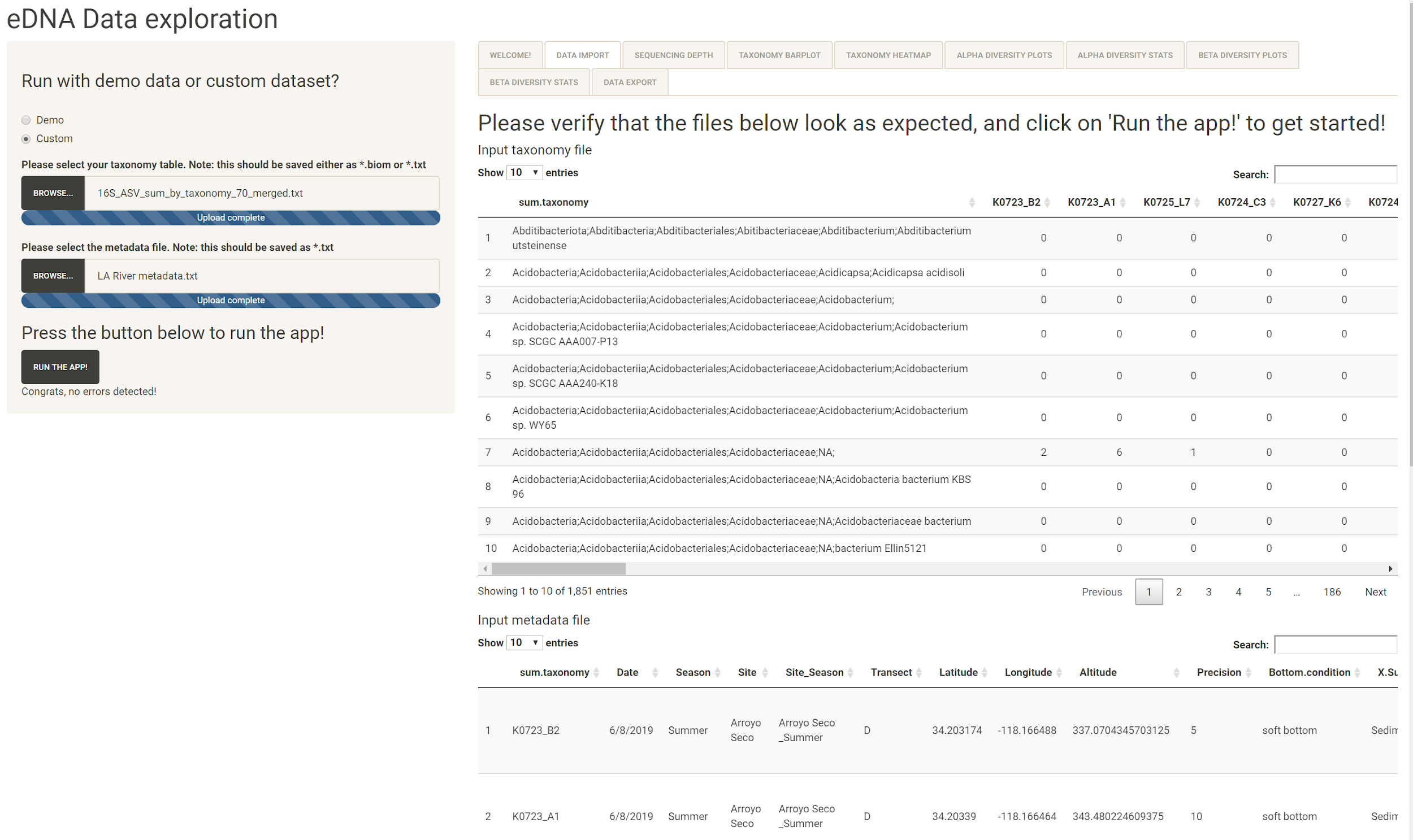


**Figure 1**: Uploading a custom dataset to Ranacapa.

**STEP 4.** For the taxonomy table, upload one of the files with “ASV_sum_by_taxonomy”. Select the one corresponding to the barcode that targets the taxa you are interested in. You can verify that the table imported correctly by scrolling right. To ensure correct import, the table must be saved as a “tab delimited text” file (.txt).

**STEP 5.** For the metadata file, upload the “metadata” file to the second link. You can verify that the table imported correctly by scrolling down. To ensure correct import, the table must be saved as a “tab delimited text” file (.txt).

You are now ready to “Run the app!”. If you get a message saying “Congrats, no errors detected!”, you can now click on the **SEQUENCING DEPTH** tab.

**If you did run into problems, please look at the troubleshooting guide at the end of this document for possible solutions.**

### *Software tutorial, cont’d*

**STEP 6.** Once you switch to the **SEQUENCING DEPTH** tab, select “none” under “Choose whether you would like to pick a custom rarefaction depth, or whether samples should be rarefied to the minimum number of sequences in any single sample” (black arrow, **Figure 2**). Wait for the figure to load (you may need to scroll down to see it).

**QUESTION 1.** Do these curves look familiar? How are they similar to species accumulation curves? How are they different?

**STEP 7.** Explore the options available to you here. You can select a variable that is of interest to you to group the curves (e.g location, date, pH, etc) by selecting from the “Select the variable” dropdown menu (green box, **Figure 2**). Read the text above the figures to understand what rarefaction does and to inform yourself about the pros and cons of doing so. If you wish you can rarefy the dataset to a specific sampling depth. To do so, select the “custom” option and indicate depth of rarefraction and number of times to rarefy (blue box, **Figure 2**). We recommend moving forward with the “none” option. Be aware that whatever option you choose here will carry forward to the other tabs. You are now ready to interact with the various tools and interactive plots in the following tabs!


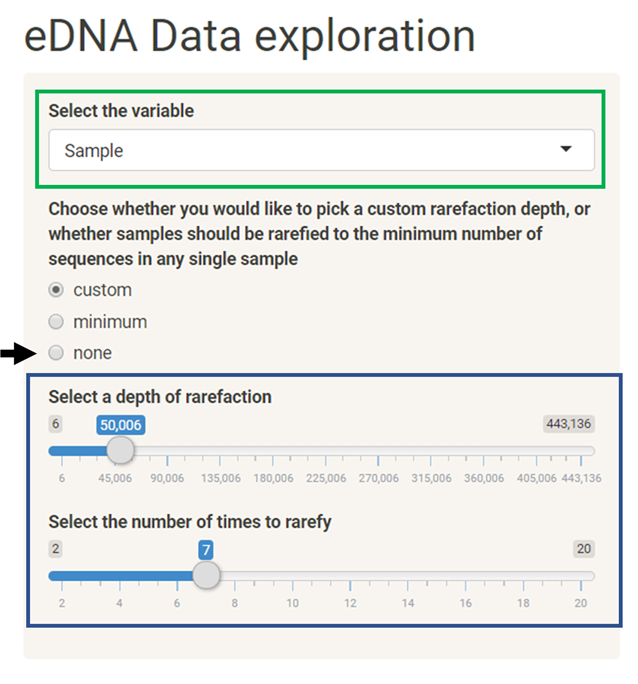


**Figure 2**: Sequencing depth options

**STEP 8.** Explore the **TAXONOMY BARPLOT**. This tool shows the taxonomy-by-sample matrix as an interactive barplot. You may have to adjust the window size to see the axis labels clearly.

**QUESTION 2.** What are the axes on this plot? We’ve discussed before that eDNA is not a good tool for measuring abundance of organisms, so what is this plot actually showing?

### *Software tutorial, cont’d*

**STEP 9.** Now move on to the **TAXONOMY HEATMAP**. This figure shows the taxon-by-sample matrix as an interactive heatmap, where the color of each cell represents the number of times a given taxon was sequenced in a sample.

**QUESTION 3.** Do you see any patterns in the heatmap in terms of true samples and negative controls (remember that you can go back to the “Data Import” tab to explore your metadata and see which samples are controls)? Is this what you expected? Why or why not?

**QUESTION 4.** You will notice that there is a line labeled “unknown”. Reflect on what you know about how taxonomic identities are assigned to DNA sequences. Can you explain in your own words what might fall under “unknown”?


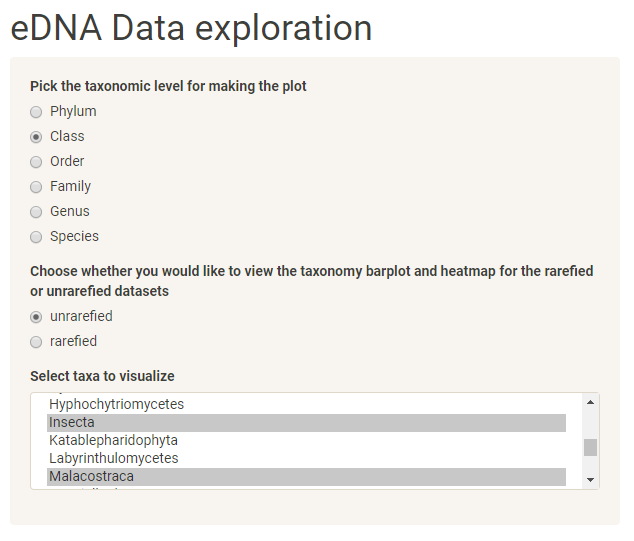


**Figure 3**. Filtering taxa of interest with the Taxonomy heatmap tool.

**STEP 10.** You can filter the taxon list by selecting or deselecting specific taxa. For example, you can select “Class” as taxonomic level for making the plot. If you were only interested in arthropod taxa, you would press and hold the “Ctrl” or “Command” button, while selecting Arachnida, Insecta, Malacostraca, Maxillopoda, etc.

**STEP 11.** It is now time to explore alpha diversity. You can do so with the **ALPHA DIVERSITY PLOTS** and **ALPHA DIVERSITY STATS.** Ranacapa allows you to pick between two different diversity metrics: observed diversity or Shannon diversity.

**QUESTION 5.** Think back to our Week 1 discussions on measuring biodiversity. Which of these two metrics takes evenness into account? Which one is a measure of species richness? Hint: read the text.

### *Software tutorial, cont’d*

**STEP 12.** The alpha diversity tools also allow you to choose different groupings for your data. Select the variables that you are most interested in exploring from the dropdown menu (**Figure 4**).


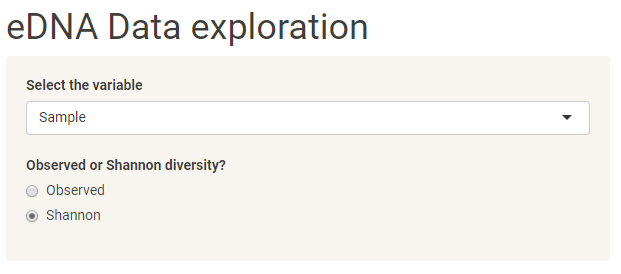


**FIGURE 4**: Exploring alpha diversity in Ranacapa

**QUESTION 6.** On the **ALPHA DIVERSITY PLOTS** page, look at the plot to determine how Shannon diversity varies depending on the season and site. In which sites (Maywood_summer, Arroyo Seco_summer or Arroyo Seco_Fall) is diversity highest? On the **ALPHA DIVERSITY STATS** page, you will be able to find the results of a statistical analysis that confirms whether your observations are statistically significant (i.e. valid) or not. If you cannot interpret the results of the ANOVA table or the Post-Hoc tests, ask your instructor to explain these to you.

**STEP 13.** You are now ready to explore beta diversity. You can do so with the **BETA DIVERSITY PLOTS** and **BETA DIVERSITY STATS.** Ranacapa will now show you a Principle Coordinate Analysis Plot (PCoA plot). The axes on these plots are hard to interpret, but the thing to keep in mind is that **samples that are more similar to each other will cluster togethe**r.

**STEP 14.** Note that points on the PCoA plot are colored according to a user-selected metadata variable. You can change the variables in the dropdown menu.

Here too, Ranacapa allows you to pick between two different diversity metrics: Jaccard dissimilarity or Bray-Curtis dissimilarity.

**QUESTION 7.** How are these two measurements different? Hint: read the text.

**QUESTION 8.** Which of these two is recommended for eDNA data and why?

**QUESTION 9.** On the **BETA DIVERSITY PLOTS** page, look at the plot to determine if community composition varies depending on the site and season. Are your points clustering by this environmental variable (i.e. by color)? On the **BETA DIVERSITY STATS** page, you will be able to find the results of a statistical analysis that confirms whether your observations are statistically significant (i.e. valid) or not. Again, ask your instructor to explain these to you if you are unsure of how to interpret these.

### *Software tutorial, cont’d*

**STEP 15.** Congratulations! You have now finished the Ranacapa tutorial. Now that you are comfortable working with this tool, try manipulating ASV tables and metadata files so that you can extract the most useful information to answer your specific question(s). For example, you may decide to:

- Add columns to the metadata file with categories of your own that better group the samples in a way that is relevant to you.
- Delete samples that are not relevant to your project.
- Delete taxonomic groups that are not relevant to your project.
- Ranacapa does not work well with numeric variables, so, if you are interested in a variable (e.g. pH) that is numerical, you might want to convert to text in a meaningful way (e.g ranges of ph between 1 and 3.9 will be “very low”, between 4 and 6.9 will be “low”, 7 will be “neutral” and so on).

While doing this, keep in mind that all the headers in the ASV table (sample names) must perfectly match the sample names listed in the metadata file for Ranacapa to run properly.

Troubleshooting guide

**Help! I cannot get past the data import tab. When I try to run the app, I get the following error: “An error has occurred. Check your logs or contact the app author for clarification.”**

1. You may have a slow connection to the server. Wait a few seconds/minutes and see if the tables or figures eventually load.
2. Make sure you have formatted your data tables correctly. **Tables must have a specific file format to be correctly imported and read by the software**. You can save them as “tab delimited text” (.txt) in Microsoft Excel, or “tab separated values” (.tsv) in Google Sheets.
3. Make sure you uploaded TWO files. The top one must be an ASV table, the second one the metadata file. If you missed one, add it and try running again.
4. If the two files are there, explore the table displays under:
   1. Input taxonomy file: The first column must be the taxonomy path in the format Kingdom; Phylum; Class; Order; Family; Genus; Species. All other columns should have read counts. The headers for these columns must be sample ID.
   2. Input metadata file: The rows in the first column must exactly match the sample IDs listed as headers in the Taxonomy file. If there are any missing or additional samples listed, or any typos that do not **EXACTLY** match between the names of the columns (input taxonomy file) and rows (metadata file), the app will not work. One way to ensure matching headers and rows between the two files is to copy the header row on the taxonomy table, and select Paste Special -> Transpose to the first column of the metadata table.

**The sequencing depth plots will not load and I get a message that says: “An error has occurred. Check your logs or contact the app author for clarification”**

Did you get a message in the previous tab (Data import) saying “Congrats, no errors detected!”. If you did not, go back and make sure the files import correctly. If you did, look in the lower right

### *Software tutorial, cont’d*

corner. If it looks like the plots are still loading, please be patient. Otherwise, refresh the page and start over.

**I have chosen a variable but no plots are displayed and I get an error message that says “An error has occurred. Check your logs or contact the app author for clarification”**

Have you selected a variable with numerical values? Ranacapa does not know how to work with these. Try editing the metadata file, converting numerical values into text values. For example you can either spell out the number (“nine” instead of “9”), or you can categorize values (“low” for values between 1-5, “medium” for values between 6-10 and so on).

### *Oxford-Style debate*

**Week 6 Day 2 In Class Activity - Oxford-Style Debate Research:**

**Should Wolves be Encouraged to Return to California?**


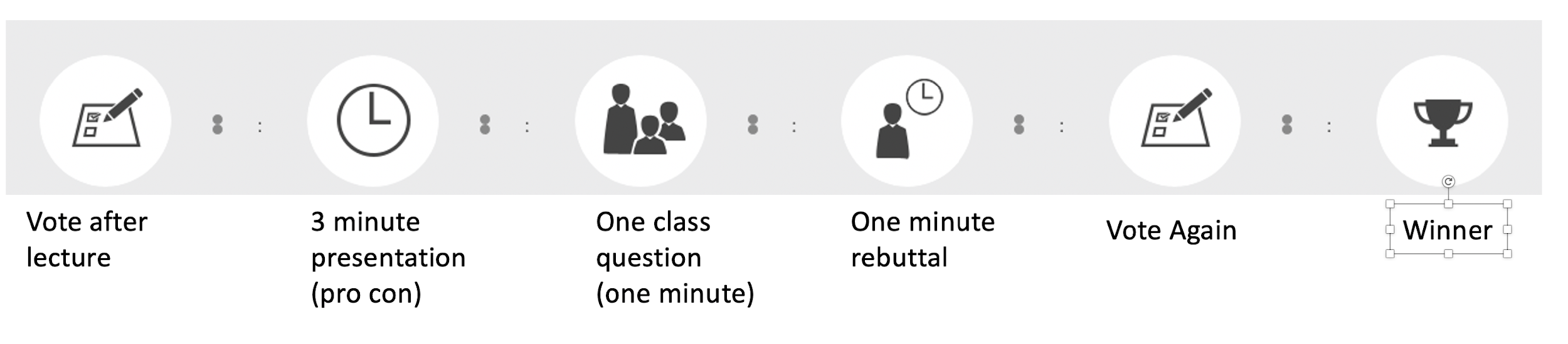


Research for debate in class today and as homework, actual debate performance next Tuesday (May 12)

There are **three main sections** for the debate on Tuesday that you need to research today:

1. Introductory arguments – Two or three main points for each stakeholder group (see below). You need to present these points concisely with supporting information and cite original sources. Discuss the merit and robustness of the supporting data.
2. Q&A Session: After introductory arguments are made, **we will ask one or two**  **questions** to both pro and con teams (possible questions listed at the end of the worksheet). You should be prepared to answer the ones associated with your stakeholder group.
3. Provide a one-minute rebuttal to critical points made by the opposition or audience.

**Everyone in your group must fairly participate in the debate. Decide who in your group will be in charge of presenting oral arguments on Tuesday, those who will not present could have more research duties, for example. You can use primary literature, news articles, policy documents, and other media to compile your argument.**

| Student Name | UCSC or UCLA | Email Address | Group Role |
| --- | --- | --- | --- |
|  |  |  |  |
|  |  |  |  |
|  |  |  |  |
|  |  |  |  |
|  |  |  |  |

### *Oxford-Style debate, cont’d*

Stakeholder Groups: There will be two pro and con sides for each group. We will split into two large breakout room groups on Tuesday and hold two parallel debates to keep numbers manageble.

-Ranchers

-Hunters

-Environmentalists

-Scientists

-Government & Industry

As you prepare your argument, you should think about:

- The other side’s argument- how can you rebut their major points?
- Who are the other stakeholders in this debate?
- How can science bolster your argument? eDNA?
- How strong is the science on either side?
- Keeping track of and citing your sources

**Your stakeholder group, side, and Thursday breakout room number (1 or 2): Ranchers, Pro, 1**

**Outline of your major points (your stakeholder group and side):**

**Outline of opposition major points (your stakeholder group, opposing side):**

**Scientific evidence that bolsters your argument. What additional scientific techniques (i.e. eDNA!) could you use? Also list some evidence that rebuts your argument to prepare for the opposing side’s views.**

**Sources used/cited:**

**Eight possible questions for the Q&A. We will ask one or two of these, and both pro and con sides will be required to provide an answer. Some stakeholder groups will have more expertise to answer some questions than others, provide an outline of an answer if your stakeholder group is listed in parentheses:**

There are other states where wolves have habitat. Is California really a good place for wolves to be re-established? Why or why not? (All groups)

### *Oxford-Style debate, cont’d*

What ecological impacts do you expect that wolves will have if they are established in California? What other species could benefit or be negatively impacted? (Hunters, Environmentalists, Scientists)

I live in a town in the re-introduction area and I’m scared of encountering wolves close to where I live. Should urban residents be concerned about encountering wolves as compared to other wild animals? (Environmentalists, Gov & Industry)

Research shows that the presence of wolves has an impact on the behavior of deer and other prey species, so that they move to different areas to avoid wolves. Deer and other large ungulate species are the primary food source of wolves. I’m a hunter in N. California. I rely on venison for food, and my hunting license fees contribute to funding the California Fish and Wildlife Service. I am concerned that wolves will negatively affect both the abundance and presence of deer in hunting grounds. What information and/or policies (if any) could address my concerns? (Hunters, Gov & Industry)

In 2015, it is thought that a wolf from the Shasta Pack killed and ate a calf from a ranch in N. California, and then the pack was not seen together after that. It is rumored that multiple wolves from the pack were shot and killed by ranchers. Can human conflicts like these be prevented? (Ranchers)

I’m a rancher in N. California. I don’t want to kill wolves but would consider it if I felt my property and livelihood were being threatened. In this case should killing a wolf that we know is taking livestock be legal? What strategies (if any) could be put in place to prevent wolves from coming onto my property? What compensation (if any) could be done if wolves take one of my animals? (Ranchers, Gov & Industry)

Maintaining genetic diversity in re-introduced wolf populations has been a problem in other wolf restoration programs like on Isle Royale and in Yellowstone. Do you anticipate that this could also be a problem in California? How can we monitor California wolf populations’ genetic diversity? What methods are available and how feasible are they? (Scientists)

For wolf reintroduction programs, wolves are often translocated from other packs into a new area. Is this justified? Why or why not? (Environmentalists, Scientists)

**Download a copy of this document as a Word document or PDF and upload individually to Canvas or CCLE before Tuesday. You can continue to work on research over the weekend if you do not have time to complete during class today – again make sure that work is evenly split between your group members, and discuss any additional research you do outside of class with the presenters in your group so they are prepared for Tuesday.**

## **Google Form**

Example: https://forms.gle/MZkqGZvMXAEWdxrz7
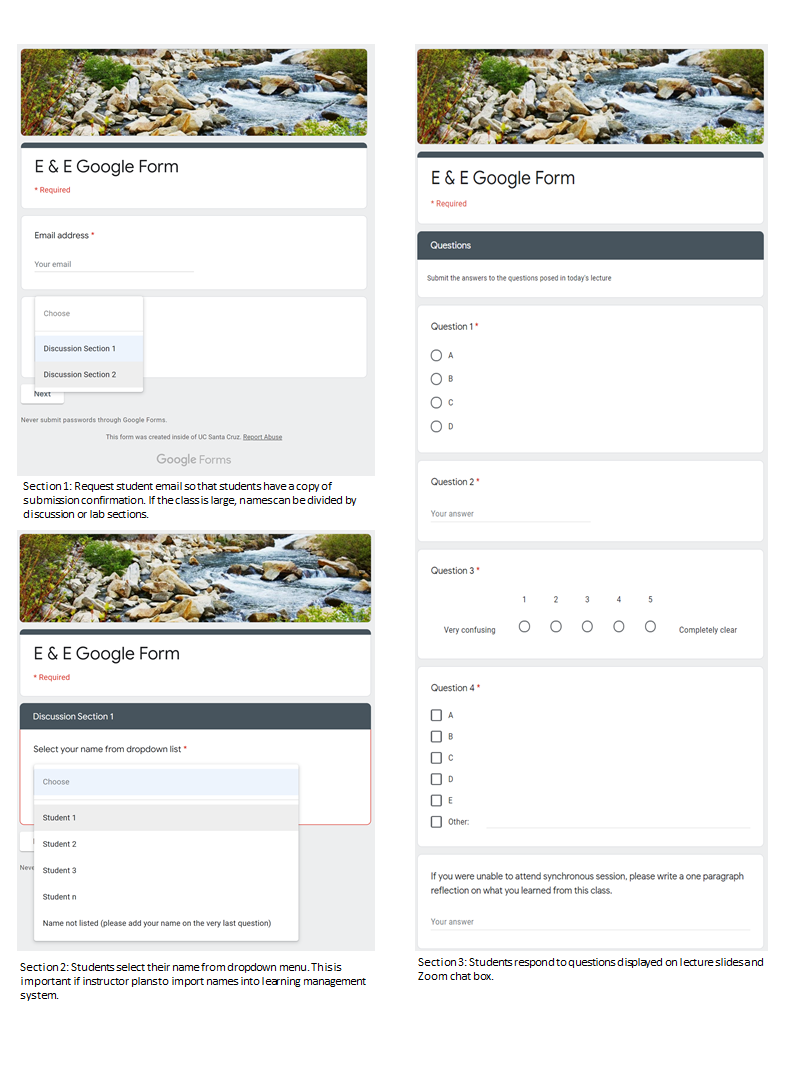

Supplement: Supplementary file 1 — Appendix S1 [file ECE3-10-12620-s001.docx]
